# Ferulic Acid Ameliorates Alzheimer’s Disease-like Pathology and Repairs Cognitive Decline by Preventing Capillary Hypofunction in APP/PS1 Mice

**DOI:** 10.1007/s13311-021-01024-7

**Published:** 2021-03-30

**Authors:** Ni-Ya Wang, Jin-Nan Li, Wei-Lin Liu, Qi Huang, Wen-Xing Li, Ya-Hong Tan, Fang Liu, Zi-Hua Song, Meng-Yue Wang, Ning Xie, Rong-Rong Mao, Ping Gan, Yu-Qiang Ding, Zhi Zhang, Bao-Ci Shan, Li-Dian Chen, Qi-Xin Zhou, Lin Xu

**Affiliations:** 1 CAS Key Laboratory of Animal Models and Human Disease Mechanisms, and KIZ-SU Joint Laboratory of Animal Model and Drug Development, and Laboratory of Learning and Memory, Kunming Institute of Zoology, the Chinese Academy of Sciences, Kunming, 650223 China; 2 Kunming College of Life Sciences, University of the Chinese Academy of Sciences, Kunming, 650223 China; 3 The Academy of Rehabilitation Industry, Fujian University of Traditional Chinese Medicine, Fuzhou, 350122 China; 4 Key Laboratory of Nuclear Analysis Techniques, Institute of High Energy Physics, the Chinese Academy of Sciences, Beijing, 100049 China; 5 CAS Key Laboratory of Brain Function and Disease, Hefei National Laboratory for Physical Sciences At the Microscale, University of Science and Technology of China, Hefei, 230027 China; 6 State Key Laboratory of Innovative Natural Drugs and Traditional Chinese Medicine Injections, Qingfeng Pharmaceutical Corporations, Ganzhou, 341000 China; 7 Kunming Medical University, Kunming, 650500 China; 8 State Key Laboratory of Medical Neurobiology and MOE Frontiers Centre for Brain Science, Institutes of Brain Science, Fudan University, Shanghai, 200032 China; 9 Mental Health Institute, the Second Xiangya Hospital of Central South University, Changsha, 410008 China; 10 CAS Centre for Excellence in Brain Science and Intelligent Technology, Shanghai, 200031 China

**Keywords:** Alzheimer’s disease, APP/PS1 mouse, Aβ plaque, Hippocampus, Endothelin-1, Ferulic acid (FA)

## Abstract

Brain capillaries are crucial for cognitive functions by supplying oxygen and other nutrients to and removing metabolic wastes from the brain. Recent studies have demonstrated that constriction of brain capillaries is triggered by beta-amyloid (Aβ) oligomers via endothelin-1 (ET1)-mediated action on the ET1 receptor A (ETRA), potentially exacerbating Aβ plaque deposition, the primary pathophysiology of Alzheimer’s disease (AD). However, direct evidence is still lacking whether changes in brain capillaries are causally involved in the pathophysiology of AD. Using APP/PS1 mouse model of AD (AD mice) relative to age-matched negative littermates, we identified that reductions of density and diameter of hippocampal capillaries occurred from 4 to 7 months old while Aβ plaque deposition and spatial memory deficit developed at 7 months old. Notably, the injection of ET1 into the hippocampus induced early Aβ plaque deposition at 5 months old in AD mice. Conversely, treatment of ferulic acid against the ETRA to counteract the ET1-mediated vasoconstriction for 30 days prevented reductions of density and diameter of hippocampal capillaries as well as ameliorated Aβ plaque deposition and spatial memory deficit at 7 months old in AD mice. Thus, these data suggest that reductions of density and diameter of hippocampal capillaries are crucial for initiating Aβ plaque deposition and spatial memory deficit at the early stages, implicating the development of new therapies for halting or curing memory decline in AD.

**Supplementary Information:**

The online version contains supplementary material available at 10.1007/s13311-021-01024-7.

## Introduction

As the population ages, Alzheimer’s disease (AD) has led to a huge and increasing socioeconomic burden on societies worldwide [1]. Previous studies have established that Aβ plaque deposition is the primary pathophysiology of AD [2–10], but the Aβ-directed therapies have failed in halting or curing the progressive decline of memory and other cognitive functions in AD patients [11]. This arises a critical question of which mechanisms of action relevant to aging could work for the therapy.

Aging is the primary factor associated with vascular changes such as reduced cerebral blood flow (CBF) and particularly correlated with the prevalence of AD [1]. Previous studies have demonstrated that reduced CBF or brain hypoperfusion is correlated with memory decline in AD [12–20]. Relatively, less attention has been paid to the changes in brain capillaries in AD patients [21, 22] or rodent models [23, 24]. A recent study has further identified an overall reduction of capillary diameter in the hippocampus by using a high-resolution mapping technique [25] in aged APP/PS1 mice. Furthermore, the soluble Aβ oligomers cause capillary constriction via the ET1-mediated ETRA activation [26], leading to brain hypoperfusion [27, 28]. Since brain hypoperfusion likely increases the accumulation and/or decreases the clearance of Aβ [29–33], this could form a feedforward cycle of the hypoperfusion-Aβ aggregation-more hypoperfusion to initiate Aβ plaque deposition. Thus, we assumed that reduced density and/or diameter of the capillaries in the hippocampus could be the earliest events relevant to aging and also for the pathophysiology of AD because cognitive functions in early AD are mostly restricted to the impairment of episodic memories [34], for which the hippocampus is well known to play a crucial role [35].

However, direct evidence for a causal involvement of hippocampal capillaries in the pathophysiology of AD remains still lacking [36]. To address this question, we used AD mice and age-matched negative littermates (WT mice) from 3 to 12 months old. We found that reduced density and diameter of hippocampal capillaries were occurring from 4 to 7 months old, but Aβ plaque deposition, spatial memory deficit, and aggregative microglial cells were all present at 7 months old in AD mice. The injection of ET1 into the hippocampus as a proof of concept for hippocampal hypoperfusion caused early Aβ plaque deposition at 5 months old in AD mice. In contrast, FA is a natural compound able to produce antioxidant and anti-inflammation actions [37] or inhibition of Aβ aggregation [38]. We found that FA could bind on the ETRA to counteract ET1-mediated vasoconstriction, and the treatment of FA for 30 days prevented reductions of density and diameter of hippocampal capillaries as well as ameliorated Aβ plaque deposition, increased aggregative microglial cells, and spatial memory deficit. Thus, we conclude that the changes of hippocampal capillaries are probably crucial for initiating Aβ plaque deposition and spatial memory deficit at the early stages, implicating the development of new therapies for memory decline in early AD.

## Materials and Methods

### Animals

The male APP/PS1 transgenic mice and their negative littermates [B6. Cg-Tag (APPswe, PSEN1dE9) 85Dbo/Mmjax, from the Model Animal Research Centre of Nanjing University, Nanjing, China], and C57 mice (from Animal Center of Kunming Medical University, Yunnan, China) were used. Animals were group-housed (4–5 littermates) in ventilated cages with free access to water and food, a 12/12-h light/dark cycle, and a thermoregulated environment. The age and number (N) of these animals were described in the “Results” sections while *n* represented the number of measured sections or slices. All experimental procedures were approved by the Institutional Animal Care and Use Committee of Kunming Institute of Zoology, the Chinese Academy of Sciences, Kunming 650223, China (SMKX-2015019).

### Ferulic Acid Treatment

Some AD mice at 6 months old were subjected to ferulic acid (FA) (Aladdin, Shanghai, China) treatment for 30 days via their daily drinking water from the water bottles placed on homecage (4–5 mice/cage). We measured the body weight of each mouse and got the average daily water intake per cage for 3 days before drug administration. In our experimental conditions, each mouse drank about 3.5–5 mL per day. Then, we prepared the drinking water with FA according to the body weight and the average water intake per day. After starting FA administration, the water bottle was changed twice a week when the drinking amount of each cage was measured. Based on these parameters, the average amount of FA consumption for each mouse was estimated to be about 20 mg/kg/day. The vehicle control in AD or WT mice at the same age took the normal drinking water without FA.

### The Morris Water Maze

The water maze consists of a 120-cm-diameter circular white plastic tank (Med Association, USA) filled with warm water (21–22 °C), and the polyethylene plastic particles are used to obscure a hidden platform (10 cm in diameter) underwater. The experimental procedures used here are like those previously described [39–42]. Briefly, the Morris water maze test includes spatial learning for consecutive 5 days and then 24 h later a probe trial for testing spatial memory. In the spatial learning task, each animal was gently released into the water maze by facing the wall of the water tank, from four different quadrants in four training trials per day, with a 10-min intertrial interval, and allowed to find the hidden platform in 60 s. If a mouse found the hidden platform within 60 s, it allowed staying on the platform for 10 s. If a mouse failed to escape to the hidden platform in 60 s, it was guided on to and stayed on the platform for 15 s. The mean time in escaping onto the hidden platform was used to score the spatial learning. The probe trial 24 h after the final training without the hidden platform was used to test spatial memory. In this probe trial, each mouse was released into the water maze also by facing the wall of the water tank, from the diagonal quadrant of the original hidden platform location, and allowed a free swim for 60 s. The time spent in the target quadrant where the hidden platform was previously located was used to score spatial memory. All the data in the Morris water maze test were recorded and analyzed by using the EnthoVision 8.0 program (from Noldus, Beijing, China).

### Immunohistochemistry

The techniques used here were like those described previously [43, 44]. Mice were anesthetized with pentobarbital sodium (80 mg/kg, i.p.) and transcardially perfused with phosphate-buffered saline (PBS) followed by 4% paraformaldehyde (PFA). Then, the brains were harvested and post-fixed in 4% PFA at 4 °C overnight and dehydrated in 30% sucrose with PBS at 4 °C for 48 h. The brains were sectioned (coronal sections in 30 μm thickness) by using a vibratome (Leica Biosystems, German). The brain sections were stained with antibodies. Besides, for capillary staining (Collagen IV), mice were only transcardially perfused with PBS, and then post-fixed in 4% PFA at 4 °C for 8 h, but the other steps were the same.

For immunofluorescence, we used the free-floating immunohistochemistry method. The sections were first washed three times and then blocked in a 0.01 M PBS containing 5% BSA and 0.3% Triton X-100 for 1 h and followed the incubation with the primary antibody overnight at 4 °C. The antibodies used were as follows: anti-Aβ (6E10, 1:1000, sig-39300, Covance), (D54D2, 1:1000, 51374, Cell Signaling), Iba1 (1:1000, 019-19741, Wako), GFAP (1:1000, ab53554, Abcam), and collagen IV (1:500, ab6586, Abcam). Next, the slices were washed three times in PBS and followed by 2-h incubation with the fluorescent secondary antibody (1:1000, Life Technologies) at room temperature. Finally, these slices were washed three times and then followed by mounting with 4′,6-diamidino-2-phenylindole (DAPI) staining, and cover-slipping on the microscope slides. Images were acquired by using a confocal microscope (Nikon A1 or Olympus FV3000).

DAB (diaminobenzidine) immunostaining was performed on 30-µm sections. The sections were placed into 0.3% hydrogen peroxide to inhibit endogenous peroxidase for 10 min, and then washed three times and blocked in a 0.01 M PBS containing 5% BSA and 0.3% Triton X-100 for 1 h and followed the incubation with the primary antibody Iba1 (1:1000, 019-19741, Wako) overnight at 4 °C. Next, the sections were washed three times in PBS and followed by 1 h incubation with biotinylated secondary antibody (1:1000, A0279, Beyotime Biotech) and 30-min incubation with Streptavidin/HRP (1:250, SE068, Solarbio) at room temperature. DAB Horseradish Peroxidase Color Development Kit (P0202, Beyotime Biotech) was used for immunohistochemical color development. Images were acquired by using an optical microscope (Olympus).

Quantification of the confocal images was performed as follows: images were acquired throughout the hippocampus and the cortex from three to six nonadjacent sections (~180 µm apart) per animal. The images were analyzed using the ImageJ software (NIH). The “analyze particles” function was used for the positive area measurement, and a set threshold was used for both control and experimental groups. For capillary diameter measurement, a line was drawn in ImageJ across the capillary perpendicular to its axis and the diameter of the capillary was measured. We measured 3 points per capillary and 10 capillaries per section for each animal.

### Thioflavin S Staining

Thioflavin S (Ths) staining was used to label the Aβ plaques. Briefly, the brain sections were washed three times and then stained with Ths (0.05 mg/mL) (T1892, Sigma-Aldrich) in dark for 10 min and followed by two washes with 50% ethanol and PBS. The sections were mounted and imaged by using the confocal microscope.

### Surgery and ET1 Injection

Using the techniques like those described previously [45, 46], the surgery was carried out under pentobarbital sodium (80 mg/kg, i.p, Sigma-Aldrich) anesthesia, the body temperature (37 ± 0.5 °C) was maintained through a heating pad, and the scalp was shaved and sterilized with povidone-iodine and 70% ethanol. Vehicle or ET1 was injected into the hippocampal CA1 region by using a stereotaxic apparatus (RWD Life Sciences, China). Under the surgery conditions, glass micropipettes were positioned to the targeted area by using the stereotaxic coordinates: dorsal CA1 regions in the hippocampus (AP, − 2.0 mm, ML ± 1.5 mm, DV − 1.7 mm). Infusion of vehicle or ET1 (1 μL, 1 μg/μL, E7764, Sigma-Aldrich) by using glass micropipettes was driven by a syringe pump (Micro 4, USA) at a speed of 0.1 μL/min. These animals were housed in an isolated room for recovery and experiment.

### Lectin Perfusion

After being anesthetized with pentobarbital sodium (80 mg/kg, i.p., Sigma-Aldrich), the mice were injected with fluorophore-conjugated tomato lectin (DL-1177, Vector Laboratories) (0.5 mg/mL, 100 μL) via intracardiac. Then the animals were perfused, and the brain was removed and sectioned for microscope imaging.

### Ferulic Acid-Biotin Injection and Immunostaining

Ferulic acid-biotin (FA-biotin) was synthesized by the Shureli Biopharma company (Kunming, China). FA-biotin (1 μL, 10 μg/μL) and biotin (1 μL, 5 μg/μL, Sigma-Aldrich) were injected into the hippocampus at a speed of 0.1 μL/min as described in the above. After the injection for 40 min for allowing the drug to be fully diffused, the mice were sacrificed for immunostaining. As described above, the sections were incubated with the primary antibody overnight at 4 °C. The antibody used was anti-ETRA (1:800, E9780, Sigma-Aldrich). Next, the slices were washed three times in PBS and followed by 2-h incubation with the secondary antibody (1:1000, Life Technologies) and Streptavidin-Alexa Fluor™ 488 conjugate (1:1000, S11223, Invitrogen) at room temperature. Finally, these slices were washed three times with PBS, followed by mounting with DAPI and cover-slipping on the microscope slides. Images were acquired by using a confocal microscope (Nikon A1 or Olympus FV3000).

### Enzyme-Linked Immunosorbent and β-Secretase Activity Assays

The hippocampus samples were homogenized in ice-cold RIPA Lysis Buffer (Beyotime Biotech), supplemented with the PMSF, protease, and phosphatase inhibitor. The concentrations of Aβ 1–40 and Aβ 1–42 in the brain extracts were measured by using an enzyme-linked immunosorbent assay (ELISA) according to the manufacturer’s instructions (R&D Systems, DAB142, DAB140B).

The β-site of the APP Cleaving Enzyme 1 (BACE1) activity in the fresh brain tissues was measured by using the β-Secretase Activity Fluorometric Assay Kit, according to the manufacturer’s instruction (Biovision, K360-100).

### Western Blot

The paradigm used was like those described previously [40, 47]**.** The hippocampal tissues of the mice were homogenized in ice-cold RIPA Lysis Buffer (Beyotime Biotech) supplemented with the PMSF (Selleck) and protease inhibitor (Millipore). The homogenates were centrifuged at 1000*g* for 15 min at 4 °C. Twenty μL of each sample being stored for BCA assay and the rest was mixed with 4 × SDS loading buffer (250 mmol Tris–HCl, pH 6.8, 20% β-mercaptoethanol, 4% SDS, 0.004% bromophenol blue (wt/vol), 40% (vol/vol) glycerol) in a 3:1 ratio, heated at 80 °C for 15 min. Each sample was run on an SDS-PAGE (10% acrylamide) and transferred to a PVDF membrane (Millipore). The membranes were blocked for 1 h with TBST (0.9% NaCl, 10 mM Tris, 0.1% Tween-20, PH7.4) containing 5% BSA on an orbital shaker at room temperature, and then incubated the primary antibody overnight at 4 °C (anti-amyloid precursor protein, 1:5000, A8717, Sigma-Aldrich; anti-tubulin, 1:10,000, CWbiotech). After three washes for 10 min each with TBST, the membranes were subsequently incubated with HRP-linked secondary antibody (goat × rabbit/mouse, HRP-linked, 1:10,000, KangChen Biotech Inc, China) for 2 h at room temperature. Immunoreactivity was detected by using Gel Imaging System (Tannon 5200 Multi) and analyzed by using the Image J software.

### Transmission Electron Microscope

The method is like a previous study [48]. The slices of the hippocampus (0.1 mm^2^) were collected from the hippocampus of WT and AD mice. After first being fixed with 3% (w/v) glutaraldehyde and 2% (w/v) paraformaldehyde, and then stained with 1.5% (w/v) potassium ferrocyanide (Sigma-Aldrich) and 1% (w/v) osmium tetroxide (Ted Pella) in cacodylate buffer (0.1 M, pH 7.4) for 40 min, followed by 1% (w/v) osmium tetroxide and 2% (w/v) uranyl acetate (SPI supplies). The tissues were dehydrated with gradient mixtures of ethanol and acetone, infiltrated by Spi-Pon 812 resin (SPI supplies). The sections with 70-nm thickness were obtained in an ultramicrotome (Leica EM UC7) and then contrasted with uranyl acetate and lead nitrate (Alfa Aesar). Finally, the tissues were detected under a Tecnai 120 kV transmission electron microscope (Thermo Fisher).

### Laser Speckle Contrast Imaging

The cerebral blood flow (CBF) was evaluated by using a laser speckle contrast imaging system (LSCI) [49] (RWD Life Sciences, China). Mice were anesthetized with a mixed air containing 1% isoflurane through a mask, and placed in a stereotaxic frame (RWD Life Sciences, China). The body temperature (37 ± 0.5 °C) was maintained through a heating pad (RWD Life Sciences, China). The scalp was then shaved and sterilized with povidone-iodine and 70% ethanol, and then incised the scalp to expose the skull, and the skull surface was cleaned and moistened with sterile saline. Real-time CBF changes were recorded using a CCD camera. The same region of interest (ROI) (red circle) was defined for all groups. For the acute effect of FA, the CBF were measured for 10 min (baseline) and after the intervention (injection of FA) continuously for 50 min. In the case of the measurements for the CBF in mice at 7 months old with the chronic FA or vehicle treatment, regional CBF was recorded throughout a 10-min period.

For measuring the blood flow of the jugular vein, C57 mice were under pentobarbital sodium (Sigma-Aldrich, 80 mg/kg, i.p.) anesthesia, because the mice were fixed on the foam board with elastic that was not fit well with the respiratory anesthesia machine (isoflurane) and then given the neck skin discission to expose the jugular vein (slightly beat, 2–4 mm in diameter), and first added saline (100 μL) directly to the exposed jugular vein and recorded for 5 min as the baseline, and then removed saline and added ET1 (1 μg/mL, 100 μL) for 10 min, and then removed the fluid and added saline (100 μL) or FA (2 mg/mL, 100 μL) for 10 min. The droplets of saline, ET1, and FA were directly given to the exposed jugular vein through a pipette. Relative blood flow changes (%) at the end of the observation period compared to the beginning of adding FA were calculated.

### Ischemic Insult and 2,3,5-Triphenyltetrazolium Chloride Staining

By using the techniques like those described previously [50, 51], the mice were injected with rose bengal (100 mg/kg, i.p. Sigma-Aldrich) and 30 min later were anesthetized with sodium pentobarbital (80 mg/kg, i.p, Sigma-Aldrich). The mice were under surgery with the body temperature (37 ± 0.5 °C) maintained by a heating pad. The photo-thrombosis model of ischemic insult was induced in the hippocampus (AP, − 2.2 mm, ML ± 1.5 mm, DV − 1.3 mm) by turning on the blue light for 20-min (473 nm, Biogene, Beijing). Then, FA was given to the mice via intraperitoneal injection (i.p.) or intragastrical administration (i.g.). The 2,3,5-triphenyltetrazolium chloride (TTC) staining was used to measure the ischemic insult of the slices taken from the brain 24 h after the induction of the photo-thrombosis model in the hippocampus.

### The Time-of-Flight Magnetic Resonance Angiography

The time-of-flight magnetic resonance angiography (MRA) was performed by using the small animal MRI 7.0 T (Bruker, Germany). Aesthesia of the mice was induced with a 1:4 mixture of oxygen and air using 2% isoflurane (RWD Life Sciences, China). During the scan, each mouse was placed prone on a small animal MRI scanning bed. The head of the mouse was placed in the surface coil special for the head of the mouse and fixed by a tooth hook and double ear rods. The body coil and surface coil were used as the exciting and receiving coil. The inner diameter of the gantry is 16 cm, and the head coil with an inner diameter of 38 mm was used. The anesthesia was maintained by the air mixed with 1.5% isoflurane through a nasal cannula of PE material during the scan. The temperature was warm and stable in the MRI scanning room. During the scanning process, the body temperature, respiratory rate, and heart rate of the mouse were monitored in real time by a physiological detector (SurgiVet V3395TPR, Smiths Medical, USA). The time-of-flight MRA scanning parameters are as follows: TR = 12 ms, TE = 3 ms, flip angle = 80°, FoV = 20 × 20 mm, averages = 4, slices = 80, slice thickness = 0.2 mm, time = 16 min 23 s 40 ms.

The cerebral blood vessels were extracted by the iterative segmentation algorithm. In brief, (1) the initial threshold T_0_ was set as the mean value of the image; (2) the image was divided into the vessel and background parts according to the threshold; (3) the mean value of the two parts was calculated: mean_vessel_ and mean_background_; (4) the new threshold *T*_*k*_ = (mean_vessel_ + mean_background_)/2. If *T*_0_ = *T*_*k*_, the process is ended; otherwise, *T*_*k*_ is set as the new threshold, and steps (2) to (4) were repeated. The newest *T*_*k*_ was used to extract the vessel part [52, 53]. After that, the vessel volume was obtained by the voxel numbers of the vessel part multiple by the volume of each voxel, and the blood vessel density was calculated by the vessel volume divided by the total intracranial volume.

### The Molecular Docking of Ferulic Acid on the Endothelin Receptor A

The crystal structure of the human endothelin receptor type-B (ETRB) and ET-1 were obtained from RCSB Protein Data Bank (https://www.rcsb.org/) with PDB ID of 5GLH. The molecule SMILES of ferulic acid (FA) was obtained from the PubChem database (https://pubchem.ncbi.nlm.nih.gov/) with PubChem CID of 445858. Because there was no structure information of the endothelin receptor type A (ETRA) in PDB, SWISS-MODEL [54] was used to conduct protein structure homology-modeling of the ETRA. The input target sequence is the human ET1 receptor isoform a precursor (NCBI reference sequence: NP_001948.1) and ran with its default parameters. The SWISS-MODEL template library (SMTL version 2018-12-13, PDB release 2018-11-23) was searched with BLAST [55] and HHBlits [56] for evolutionary related structures matching the target sequence. The template with the highest quality (PDB ID: 5GLI) has then been selected for model building. Molecular docking analysis of FA with the ETRA was carried out with AutoDock Vina 1.1.2 [57]. All docking results were visualized and analyzed by using Discovery Studio 3.1.

### RNA Sequencing and Data Analysis

The tissues from the hippocampus and the cortex of AD mice treated with drinking water containing FA (AD-FA) or AD and WT mice treated with the normal drinking water without FA were collected and stored in RNAlater (ThermoFisher) at − 80 °C. Five mice in each group were used. RNA sequencing (RNA-seq) was performed by Novogene (Beijing, China). Briefly, the total RNA was extracted by using TRIzol reagent. Sequencing libraries were generated by using NEB. Next, Ultra RNA Library Prep Kit for Illumina (NEB, USA) following the manufacturer’s recommendations and index codes were added to attribute sequences to each sample. Then, the products were purified (AMPure XP system) and library quality was assessed by the Agilent Bioanalyzer 2100 system. The clustering of the index-coded samples was performed on a cBot Cluster Generation System using the HiSeq 4000 PE Cluster Kit (Illumia) according to the manufacturer’s instructions. After cluster generation, the library preparations were sequenced on an Illumina Hiseq 4000 platform and 150 bp paired-end reads were generated.

The raw RNA-Seq data (paired-end reads) of the “fastq” format were processed with Skewer (version: 0.2.2) [58] for quality control and adapter trimming. Then, Pairfq was used (version: 0.17.0, https://github.com/sestaton/Pairfq) for reads pairing and removing the unpaired reads in each filtered paired-end file. The generated clean data with high quality were used for further analysis. Mouse reference genome and gene model annotation files (genome assembly: GRCm38.90) were downloaded from the Ensemble database (http://asia.ensembl.org/index.html). The genome index was built by using the python scripts included in the HISAT2 package (version: 2.1.0) [59, 60]. The paired-end clean reads were aligned to the reference genome by using HISAT2 and run with the default parameters. The aligned file in the “sam” format was sorted and converted to a “bam” format by using SAMtools (version 1.6) [61]. Transcripts assembly and estimating expression levels of the sorted reads were done by using StringTie (version 1.3.3b) [62] and run with the default settings. Next, the FPKM (fragments per kilobase of transcript per million fragments mapped) of each gene was calculated and summarized in the “Ballgown” package (version 2.2.0) [63].

The mouse gene annotation file was obtained by using the BioMart tool in the Ensemble database [64]. Gene annotation of the processed RNA-Seq file was made by using custom-written R scripts. There were 44 types of RNAs in the annotated expression matrix for which mRNAs were extracted (the type of “protein coding”) for further analysis. Since the data contain two brain regions from three groups, we retained a gene with a log2 FPKM expression greater than 0.1 in more than 3 samples per group to ensure the data quality. Finally, we got an expression matrix with 12,466 unique genes and 36 samples.

The R statistical software v3.4.1 was used to perform data analysis. Differentially expressed gene analysis was performed by using the empirical Bayes algorithm in the “limma” package [65] in R. Differences (up- or down-regulated) were considered statistically significant if the absolute value fold changes higher than 1.5 and the *P* values ≤ 0.05. Differentially expressed genes were calculated between AD *vs.* WT; AD-FA *vs.* AD; AD-FA *vs.* WT in both the hippocampus and the cortex. The expression profiles of the union set of differentially expressed genes in the three comparisons were showed by heatmap in the “pheatmap” package. We chose the “ward.D2” algorithm to perform hierarchical clustering for samples in different groups.

The gene sets of GO biological processes were downloaded from the QuickGO database (https://www.ebi.ac.uk/QuickGO/). We filtered the gene sets correlated to specific functions including amyloid, astrocyte, microglia, synapse, vascular, and immune-related functions, or signal pathways. We mainly focused on the expression changes in the above gene sets between AD and WT, and AD and AD-FA.

### Statistical Analysis

All data are represented as mean ± SEM. All analyses were performed using GraphPad Prism 7. Data were analyzed by using unpaired Student’s *t*-test for two independent groups, paired one for before and after comparison, and one-way or two-way ANOVA among groups followed by multiple comparisons. The significance level was set at *P* < 0.05.

## Results

### Reduction of Diameter and Density of Hippocampal Capillary Precedes Aβ Plaque Deposition and Spatial Memory Decline in APP/PS1 Mice

**Fig. 1:**
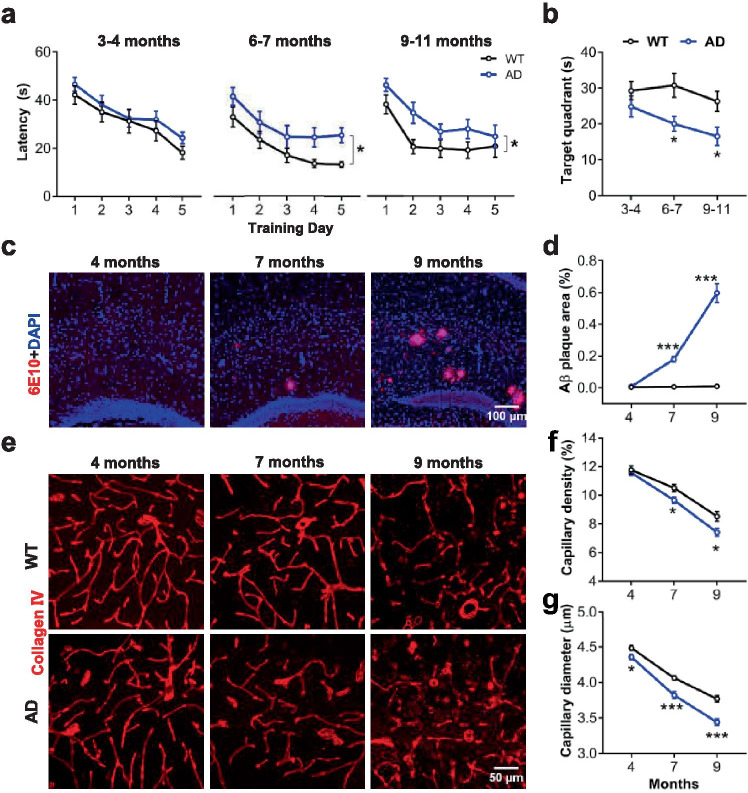
Reduced hippocampal capillaries precede Aβ plaque deposition and spatial memory deficit in APP/PS1 mice. (**a**) Spatial learning indicated by latency (s) onto a hidden platform in WT (black circle) *vs.* AD mice (blue circle) was not different until 6 to 7 months old. (**b**) Spatial memory was not different until 6 to 7 months old. (**c**) Representative images for hippocampal Aβ plaques using the antibody *6E10* in AD mice. (**d**) Aβ plaque area (%) indicated a sharp increase of Aβ plaque load since 7 months old in AD mice. (**e**) Representative images for hippocampal capillaries using the antibody *Collagen IV* in AD and WT mice. (**f**, **g**) Capillary density (%) and diameter (μm) suggested an age-dependent reduction of hippocampal capillaries in AD mice relative to WT mice. Data are presented as mean ± SEM. *P < 0.05, **P < 0.01, ***P < 0.001

We first assessed AD and WT mice using the spatial learning task of the Morris water maze [35], a type of episodic memories. We found that AD mice displayed a progressive worse decline of memory than WT mice did, as manifested by spatial learning over 5-day training and spatial memory tested 24 h after the learning, both of which were unaffected from 3 to 4 months old, but mildly impaired from 6 to 7 months old, and more impaired from 9 to 11 months old, relative to WT mice (Fig. 1a, b, *Spatial learning*; 3 to 4 months old: *N* = 14/group, *F*_(1, 26)_ = 1.074, *P* = 0.309; 6 to 7 months old: *N* = 12/group, *F*_(1, 22)_ = 7.539, *P = 0.011; 9 to 11 months old: *N* = 11 for WT, *N* = 12 for AD, *F*_(1, 21)_ = 5.838, *P = 0.024; repeated measure ANOVA. *Spatial memory*. Group, *F*_(1, 69)_ = 13.680, *P* < 0.001; age, *F*_(2, 69)_ = 2.184, *P* = 0.120; group × age, *F*_(2, 69)_ = 0.803, *P* = 0.452. Holm-Sidak’s analysis for WT *vs.* AD: 3 to 4 months old, *P* = 0.229; 6 to 7 months old, *P = 0.025; 9 to 11 months old, *P = 0.035; two-way ANOVA).

Immunostaining for Aβ plaque was then performed. AD mice showed a progressive load of Aβ plaques in the hippocampus, as demonstrated no Aβ plaques at 4 months old, but scattered Aβ plaques at 7 months old, and more Aβ plaques at 9 months old, relative to WT mice (Fig. 1c, d, 4 months old: WT, *n* = 8 (*N* = 3); AD, *n* = 9 (*N* = 3); 7 months old: WT or AD, *n* = 10 (*N* = 3); 9 months old: WT or AD, *n* = 12 (*N* = 3); group, *F*_(1, 55)_ = 110.9, *P* < 0.001; age, *F*_(2, 55)_ = 55.73, *P* < 0.001; group × age, *F*(_2, 55_) = 53.74, *P* < 0.001; Holm-Sidak’s analysis for 4 months old: WT *vs.* AD, *P* = 0.910; for 7 months old: WT *vs.* AD, ***P < 0.001; for 9 months old: WT *vs.* AD, ***P < 0.001; two-way ANOVA).

We then used collagen IV immunostaining to measure capillary changes in the hippocampus where Aβ plaques were first found. Notably, the capillaries in AD mice exhibited a progressive worse reduction than those in WT mice did, as indicated by unaltered density but reduced diameter since 4 months old, but reduction of density and diameter at 7 months old and more reduction of the both at 9 months old, relative to WT mice (Fig. 1e–g, *Capillary density*: 4 months old: WT or AD, *n* = 18 (*N* = 3); 7 months old: WT or AD, *n* = 18 (*N* = 3); 9 months old: WT, *n* = 17 (*N* = 3); AD, *n* = 16 (*N* = 3); group, *F*_(1, 99)_ = 11.61, *P* = 0.001; age, *F*_(2, 99)_ = 101.5, *P* < 0.001; group × age, *F*_(2, 99)_ = 1.664, *P* = 0.194; Holm-Sidak’s analysis for 4 months old: WT *vs.* AD, *P* = 0.589; for 7 months old: WT *vs.* AD, *P = 0.039; for 9 months old: WT *vs.* AD, *P = 0.011. *Capillary diameter*: *n* = 18 (*N* = 3)/group for each age; group, *F*_(1, 102)_ = 45.40, *P* < 0.001; age, *F*_(2, 102)_ = 187.2, *P* < 0.001; group × age, *F*_(2, 102)_ = 2.838, *P* = 0.0632; Holm-Sidak’s analysis for 4 months old: WT *vs.* AD, *P = 0.035; for 7 months old: WT *vs.* AD, ***P < 0.001; for 9 months old: WT *vs.* AD, ***P < 0.001; two-way ANOVA). This result suggests that that the earliest change is the reduced diameter of capillaries at 4 months old, and the later change is the reduced diameter and density of capillaries at 7 months old when Aβ plaque deposition and spatial memory deficit are observed.

These findings are particularly interesting because we found that changes of hippocampal capillaries could be long before the onset of Aβ plaque deposition and memory decline [66], probably consistent with the neurovascular hypotheses of AD [19, 67].

The ultrastructures associated with hippocampal capillaries from AD and WT mice aged 3 to 12 months old were also examined using transmission electron microscopy (Supplementary Fig. 1a). The extra-capillary astrocytic end feet might develop edema, and the glial pedal plate could begin mild degeneration in AD mice aged 6 months old (Supplementary Fig. 1b). Other ultrastructures such as the thickness of the perivascular basement membrane, the tight junction between the endothelial cells, and the pericyte on the outside of the capillaries were detected not a difference between AD and WT mice from 3 to 6 months old (Supplementary Fig. 1c–e).

Hippocampal microglia and astrocyte were further examined. Immunostaining of microglia showed that the total number of microglial cells was not different between AD and WT mice at 4 months old but increased in AD mice at 7 months old or 9 months old (Supplementary Fig. 2a, b). However, activated microglial cells were distributed in proximity to Aβ plaques and may release proinflammatory factors. Thus, three or more microglial cells clustered together were defined as “aggregative microglial cells” (Supplementary Fig. 2a, yellow box). We found that the area of aggregative microglial cells was increased in AD mice since the 7 months old (Supplementary Fig. 2c). The astrocytic area showed no difference between AD and WT mice at 4 or 7 months old; it was increased in AD mice at 9 months old (Supplementary Fig. 2d, e). Furthermore, the increased number of microglial cells at 7 months old was confirmed by using DAB staining (Supplementary Fig. 2f, g).

**Fig. 2:**
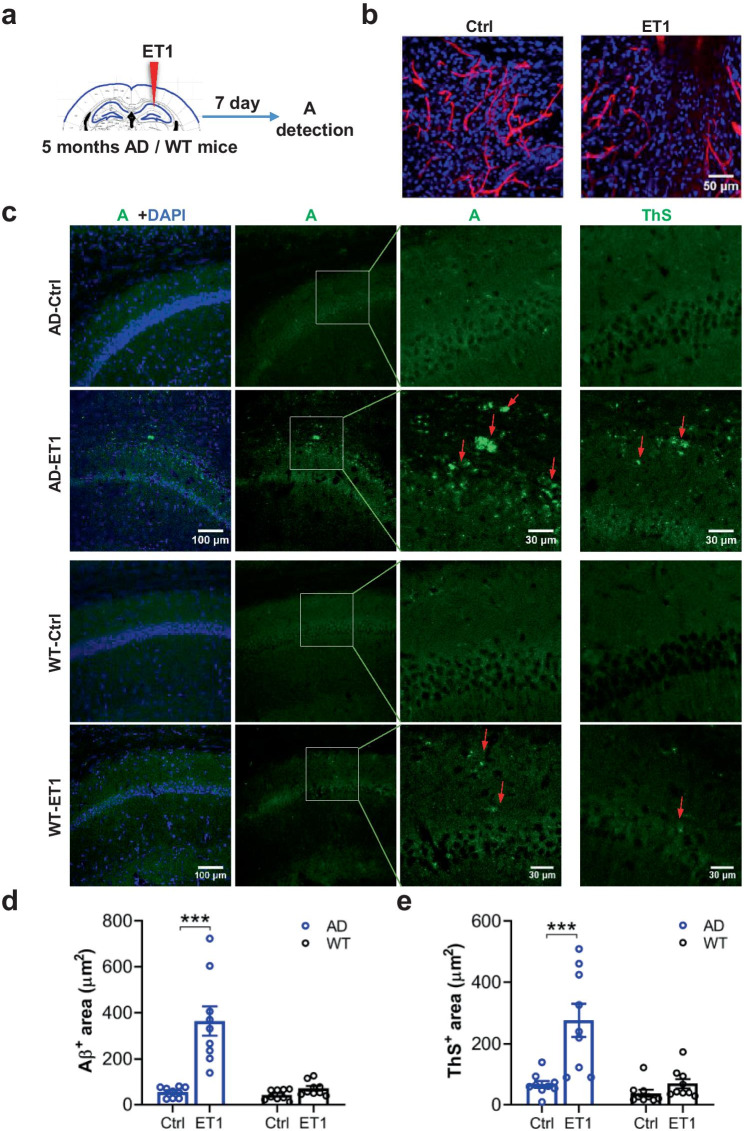
ET1 induces a tiny hypoperfusion and early Aβ plaque deposition at 5 months old. (**a**) Procedures: ET1 or vehicle injection (1 µL, 0.1 µL/min, 1 µg/µL) into one side of hippocampal CA1. (**b**) Lectin perfusion and imaging revealed a hypoperfusion insult in the ET1 group but not the vehicle group. (**c**) Representative images for Aβ plaque using the antibody *D54D2* (Aβ) or *Thioflavine S* (Ths) in AD mice. (**d**, **e**) Aβ^+^ or Ths^+^ area (μm^2^) indicated early Aβ plaque deposition in the ET1 group but not the vehicle group in AD mice, nearly undetectable in both the groups in WT mice. Data are presented as mean ± SEM. ***P < 0.001

Together, AD mice showed the early changes of the hippocampus from 4 to 7 months old: the earliest one was the reduced diameter of hippocampal capillaries at 4 months old, and the later ones were the reduced density and diameter of hippocampal capillaries, hippocampal Aβ plaque deposition, the increased number of microglial cells and increased area of aggregative microglial cells, and spatial memory deficit at 7 months old. Other changes were either not significant or later than this age.

### ET1 Induces Early Aβ Plaque Deposition

The reduced density and diameter of hippocampal capillaries are age-dependent and could have caused inconspicuous tiny hypoperfusion. This hypoperfusion can be mimicked by the injection of ET1 into the hippocampus [68]. Therefore, to address whether reduced density/diameter of hippocampal capillaries could be sufficient for initiating Aβ plaque deposition, we injected ET1 or vehicle into hippocampal CA1 region in WT or AD mice aged 5 months old (Fig. 2a) when there are no Aβ plaques at this young age. Lectin perfusion and imaging revealed a small hypoperfusion insult in WT or AD mice 7 days after the ET1 injection (Fig. 2b). Notably, the staining of Aβ plaques using either the antibody *D54D2* or *Thioflavine S* similarly revealed that ET1 caused early Aβ plaque deposition in the ET1 but not the vehicle group in AD mice. In contrast, Aβ plaques were nearly undetectable in both the ET1 and control groups in WT mice (Fig. 2c–e, *n* = 9 (*N* = 3)/group; *Aβ*^+^ *area* for Vehicle *vs.* ET1: AD mice, ***P < 0.001; WT mice, *P* = 0.347; *Ths*^+^ *area* for Vehicle *vs.* ET1: AD mice, ***P < 0.001; WT mice, *P* = 0.766; two-way ANOVA).

These results suggest that reduced density and diameter of hippocampal capillaries mimicked by the injection of ET1 into the hippocampus could be sufficient for initiating Aβ plaque deposition, consistent with the neurovascular hypotheses [19, 67], for which vascular damage can increase Aβ accumulation [29–33] or decrease Aβ clearance [67].

### FA Prevents Reduction of Both CBF and Density/Diameter of Hippocampal Capillaries in APP/PS1 Mice

**Fig. 3:**
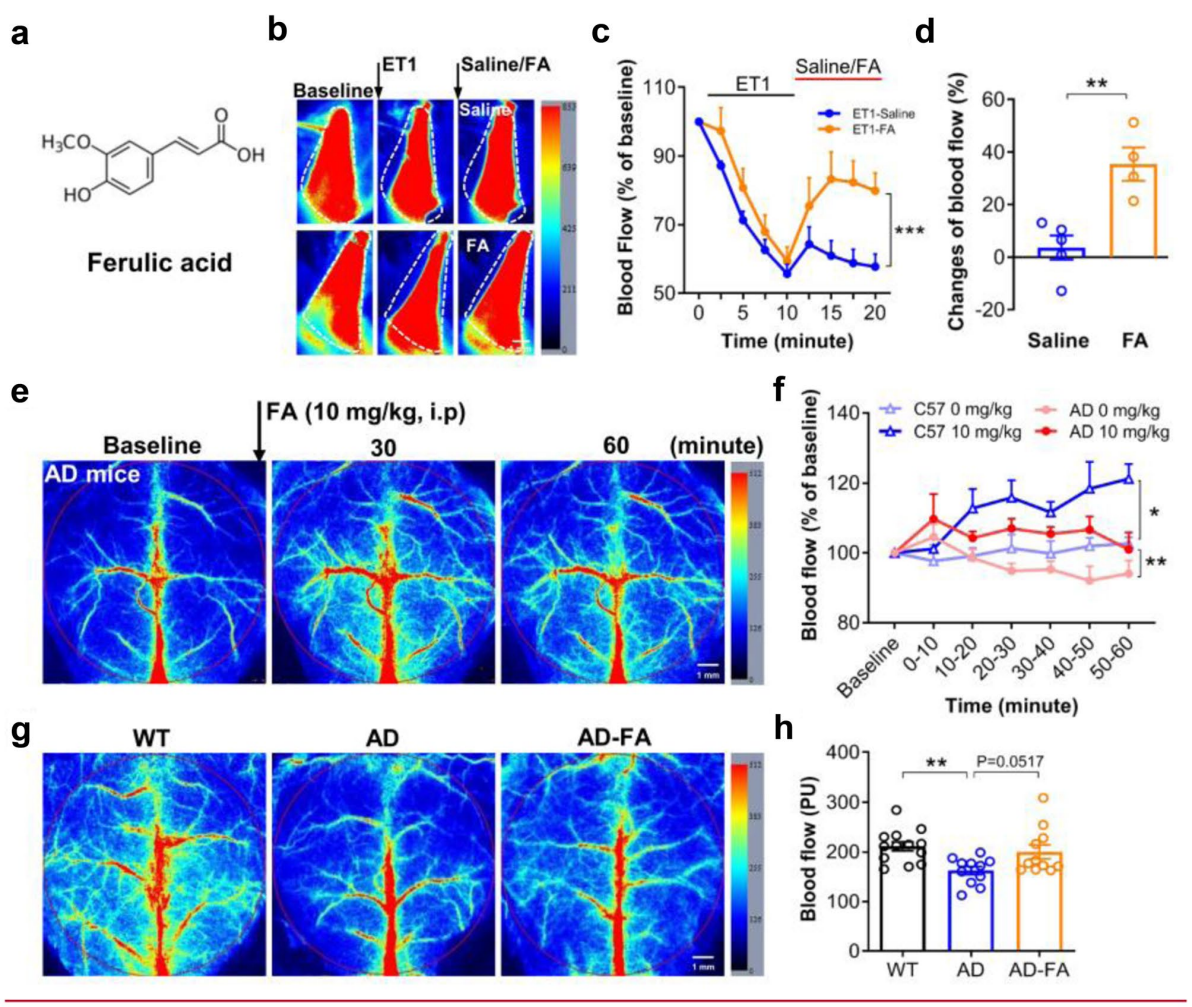
FA prevents the reduction of the CBF. (**a**) Chemical structure of ferulic acid (FA). (**b**) Experimental procedures and representative images of laser speckle imaging for the blood flows in the jugular vein. (**c**) ET1 (1 µg/mL, 100 µL) induced strong vasoconstriction as indicated by the reduction of the blood flows, while following application of FA (2 mg/mL, 100 µL) but not vehicle (Saline) induced vasodilation in C57 mice at 2 months old. (**d**) Relative changes (%) of the blood flows before and after adding FA or saline for 10 min indicated that FA counteracted the ET1-mediated action. (**e**) Representative images of laser speckle imaging in AD mice at 6 months old after intraperitoneal injection (i.p.) of FA (10 mg/kg). (**f**) The CBF was increased by FA (10 mg/kg, i.p.) in AD or C57 mice. (**g**, **h**) The FA treatment rescued the CBF reduction in AD mice that was observed in the vehicle group, relative to WT mice, by using laser speckle imaging at 7 months old. The same region of interest (ROI) (red circle) was defined for all groups

To address whether reduced density and diameter of hippocampal capillaries could be also necessary for initiating Aβ plaque deposition, we used FA (or 4-hydroxy-3-methoxycinnamic acid) (Fig. 3a), because previous studies have suggested its antioxidant or anti-inflammatory actions to be potential for treating AD [37, 38] and because we found that intraperitoneal injection (i.p.) or intragastric administration (i.g.) of FA alleviated hypoperfusion insult in the hippocampus with a dose-dependent effect (Supplementary Fig. 3). Notably, we found that FA could antagonize the ET1-mediated vasoconstriction, as examined using laser speckle imaging on the blood flow: applying ET1 evoked strong vasoconstriction; following application of FA induced vasodilation (Fig. 3b, c, ETI**-**Saline, *N* = 5; ET1-FA, *N* = 4; *F*_(1, 63)_ = 31.15, ***P < 0.001; two-way ANOVA). Compared with saline, following application of FA counteracted the ET1-mediated vasoconstriction (Fig. 3d, Saline, *N* = 5; FA, *N* = 4;* t* = 4.165, *df* = 7, **P = 0.004; Student’s *t*-test). Furthermore, molecular docking analysis revealed that FA could bind on the ETRA, the pharmacological target of ET1, with a high affinity (Supplementary Fig. 4a, b). Besides, the synthesized FA-biotin was co-localized mainly with the ETRA on the blood vessels of the hippocampus (Supplementary Fig. 4c).

**Fig. 4:**
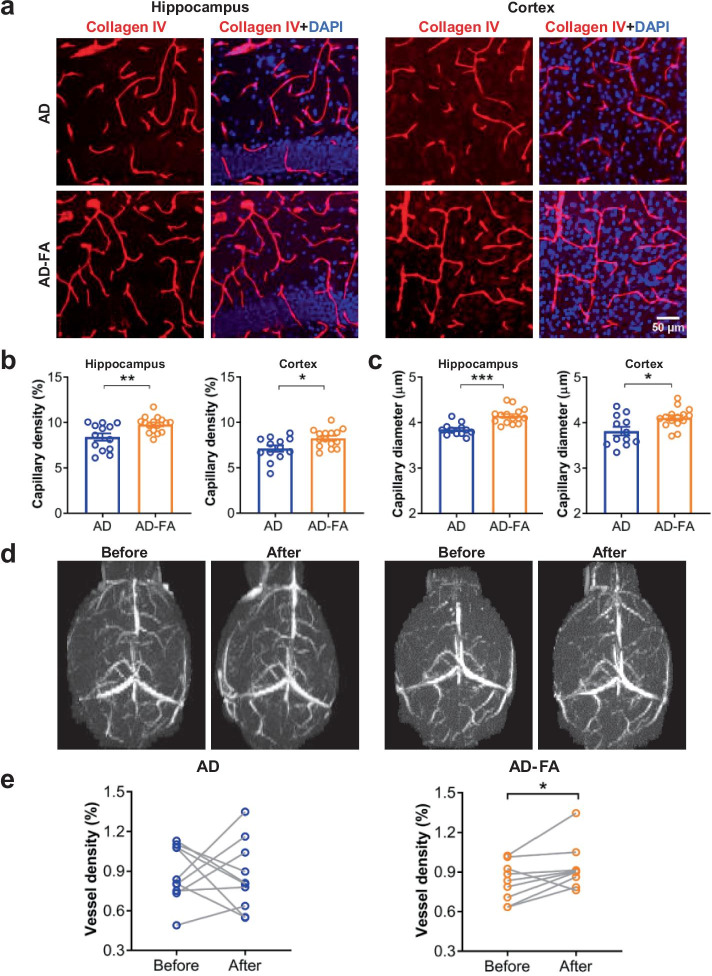
FA prevents the reduction of hippocampal capillaries in AD mice at 7 months old. (**a**) Representative images for immunostaining of the blood vessel (*Collagen IV*) in the hippocampus and cortex in AD mice after the FA or vehicle treatment. (**b**, **c**) Quantification of capillary density (%) (**b**) and diameter (μm) (**c**) in the hippocampus and the cortex suggested that the both were significantly increased by the FA treatment relative to the vehicle control. (**d**) Representative 2D time-of-flight images for blood vessels in AD mice before and after the FA treatment or vehicle control. (**e**) Comparison of the time-of-flight magnetic resonance angiography between before and after the treatment suggested that the FA treatment but not the vehicle control significantly increased the density of the whole-brain blood vessels. Data are presented as mean ± SEM. *P < 0.05, **P < 0.01

Therefore, we further investigated if FA could increase CBF in AD mice. A single treatment of FA (i.p., 10 mg/kg) induced a significant increase of CBF in C57 or AD mice (Fig. 3e, f, *N* = 4/group; *C57 mice*: dose, *F*_(1, 6_) = 9.988, *P = 0.019; Time, *F*_(6, 36)_ = 4.603, *P* = 0.001; dose × Time, *F*_(6, 36)_ = 2.348, *P* = 0.051. *AD mice*: dose, *F*_(1, 6)_ = 15.69, **P = 0.007; Time,* F*_(6, 36)_ = 1.548, *P* = 0.190; dose × Time, *F*_(6, 36)_ = 1.031, *P* = 0.421. Two-way ANOVA). Thereafter, we administered FA to AD mice chronically, started at 6 months old via daily drinking water for 30 days (averaged about 20 mg/kg/day, termed as AD-FA), while the normal drinking water without FA as the vehicle controls (AD or WT mice). CBF in AD mice with the vehicle treatment (AD, Fig. 3g) was lower than that in WT mice aged 7 months old, but this CBF reduction was prevented by the FA treatment (AD-FA, Fig. 3h, WT, *N* = 13; AD or AD-FA, *N* = 11; *F*_(2, 32)_ = 5.773, *P* = 0.007; WT *vs*. AD, **P = 0.006; WT *vs*. AD-FA: *P* = 0.738; AD *vs*. AD-FA: *P* = 0.051. One-way ANOVA followed by Tukey’s analysis). Notably, immunostaining of capillaries (Fig. 4a) showed that this 30-day FA treatment increased capillary density (Fig. 4b, *hippocampus*: AD, *n* = 13 (*N* = 4); AD-FA, *n* = 16 (*N* = 5); *t* = 3.13, *df* = 27, **P = 0.004; *cortex*: AD, *n* = 13 (*N* = 4); AD-FA, *n* = 15 (*N* = 5);* t* = 2.65, *df* = 26,*P = 0.013; Student’s *t*-test) and diameter (Fig. 4c, *hippocampus*: AD, *n* = 12 (*N* = 4); AD-FA, *n* = 15 (*N* = 5); *t* = 4.951, *df* = 25, ***P < 0.001; *cortex*: AD, *n* = 12 (*N* = 4); AD-FA, *n* = 15 (*N* = 5); *t* = 2.778, *df* = 25, *P = 0.010; Student’s *t*-test) in the hippocampus and cortex.

Furthermore, a non-intrusive method, i.e., the time-of-flight magnetic resonance angiography (MRA), was used to measure the potential changes of the whole-brain blood vessel density. AD mice were subjected to a 7-T MRA scan at 6 months old and underwent a similar paradigm of the FA or vehicle treatment for 30 days, and subjected to the 7-T MRA scan again at 7 months old. We found that the density of the whole-brain blood vessels was increased in the AD-FA group as compared with the pre-treatment baseline, but not in the vehicle-treated AD group (Fig. 4d, e, AD, *N* = 10; AD-FA, *N* = 9; *AD*: *t* = 0.208, *df* = 9, *P* = 0.839; *AD-FA*: *t* = 2.355, *df* = 8, *P = 0.046. Paired *t*-test). This finding strongly suggests that FA treatment is beneficial for increasing the density of the whole-brain blood vessels in AD mice, highly consistent with the above studies for density and diameter of capillaries.

### FA Prevents Aβ Plaque Deposition Partially

**Fig. 5:**
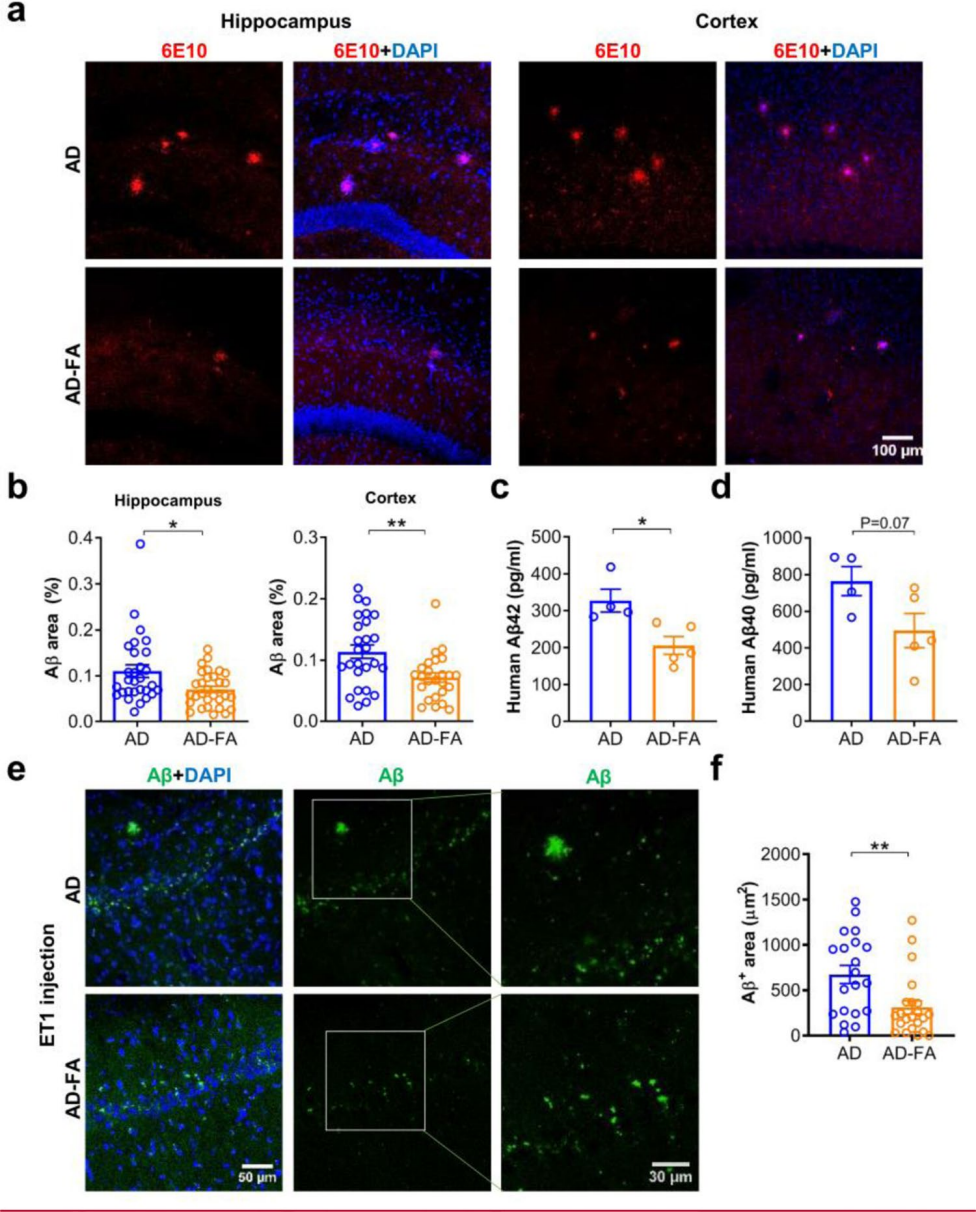
FA reduced Aβ plaque deposition in AD mice at 7 months old and ET1-induced early Aβ deposition. (**a**) Representative images for immunostaining of the Aβ antibody (6E10) in AD mice at 7 months old after the FA (AD-FA) or vehicle treatment (AD) for 30 days. (**b**) Quantification of the Aβ area (μm^2^) in the hippocampus and the cortex suggested a significant reduction of Aβ plaques by the FA treatment relative to the vehicle control. (**c**, **d**) The enzyme-linked immunosorbent assay test suggested a significant reduction of the Aβ 1–42 (**e**) and a downtrend of the Aβ 1–40 (**f**) concentration from AD mice after the FA treatment relative to the vehicle control. (**e**) Representative images for immunostaining of the early Aβ plaques (*D54D2*) initiated by the injection of ET1 into the hippocampal CA1 region in AD mice at 5 months old and underwent the ferulic acid (FA) treatment (AD-FA) or vehicle treatment (AD) for 30 days, started from the same day with the ET1 injection. (**f**) Quantification of the Aβ area (µm^2^) suggested that the FA treatment significantly reduced the ET1-induced early Aβ plaque deposition relative to the vehicle treatment

We then tested if FA could prevent Aβ plaque deposition in AD mice. Aβ plaque deposition in the hippocampus and cortex in AD mice at 7 months old was also largely prevented by the FA treatment relative to the vehicle controls (Fig. 5a, b, *hippocampus*: AD, *n* = 29 (*N* = 5); AD-FA, *n* = 30 (*N* = 5); *t* = 2.604, *df* = 57, *P = 0.011; *Cortex*: AD, *n* = 26 (*N* = 5); AD-FA, *n* = 25 (*N* = 5); *t* = 3.131, *df* = 49, **P = 0.002. Student’s *t*-test). These effects of FA were likely achieved by reducing the accumulation of Aβ in the hippocampus because the ELISA test showed a significant reduction of the Aβ 1–42 and a downtrend of the Aβ 1–40 in the hippocampus from AD mice with the FA treatment relative to the vehicle controls (Fig. 5c, d, AD, *N* = 4; AD-FA, *N* = 5; *Aβ42*: *t* = 3.145, *df* = 7, *P = 0.016; *Aβ40*: *t* = 2.136, *df* = 7, *P* = 0.070. Student’s *t*-test). Likewise, the early Aβ plaque deposition in the hippocampus, initiated 7 days after the hippocampal injection of ET1 at 5 months old, was persistent up to 6 months old with the vehicle treatment, but that was partially prevented by the FA treatment, reduced to a level about 50% of the vehicle controls (Fig. 5e, f, AD, *n* = 20 (*N* = 7); AD-FA, *n* = 22 (*N* = 7); *t* = 2.987, *df* = 40, **P = 0.005. Student’s *t-*test). These reduced Aβ levels in the hippocampus were not attributable to a change of APP expression because western blot of the hippocampal tissues from AD mice showed no significant changes in the total levels of APP protein between the FA and vehicle groups (Supplementary Fig. 5a). Also, the β-site of the APP cleaving enzyme 1 (BACE1) activity, measured by using the β-secretase activity fluorometric assay kit, was increased in the hippocampus from AD mice with the vehicle treatment but was backed to the WT levels by the FA treatment (Supplementary Fig. 5b). Consistent with earlier reports, FA or FA-based hybrid treatment reduced BACE1 expression and β-secretase activity in AD mice or cultured APP-overexpressing murine neuron-like cells [69]. The possible mechanism is that FA rescued the hypoxia-up-regulated BACE1 activity [29] and/or inhibited the NFκB-dependent transcription of BACE1 [70]. It has been also reported that FA may directly interact with BACE1 and inhibit its activity [71].

We next performed co-immunostaining of Aβ plaque deposition with microglia in the hippocampus from AD mice at 7 months old after the 30-day FA or vehicle treatment. No significant differences were found in the total number of microglial cells, but the area of the aggregative microglial cells, characterized by more than 3 cells aggregated proximity to the Aβ plaques, was significantly reduced after the FA treatment relative to vehicle control (Supplementary Fig. 6a–c). In contrast, immunostaining for astrocyte showed that the FA treatment produced no significant effect on the astrocytic area (Supplementary Fig. 6d–f), due to no changes at this age (see 7 months old in Supplementary Fig. 2d, e).

**Fig. 6:**
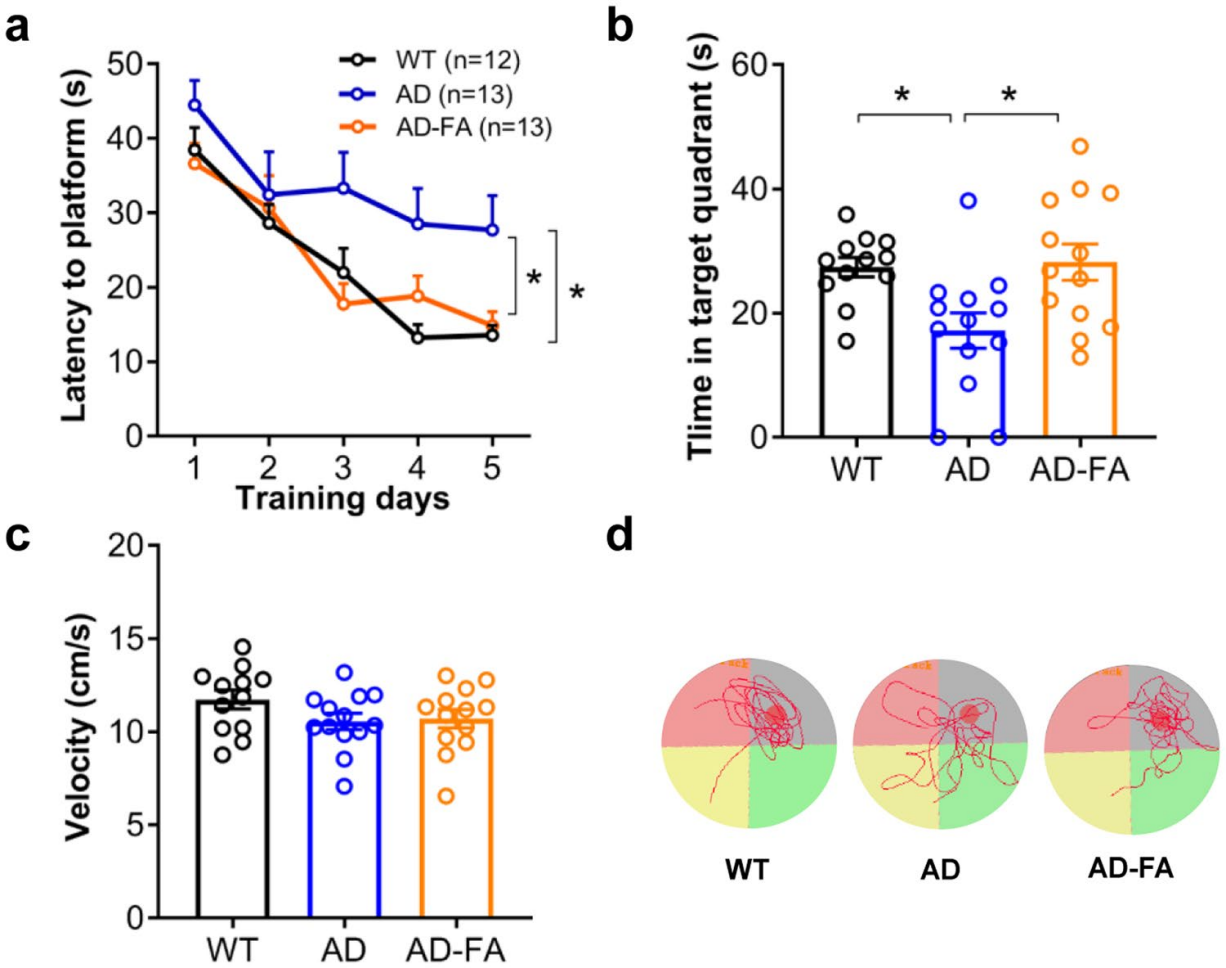
FA repairs spatial memory deficit in AD mice at 7 months old. (**a**) Spatial learning, as indicated by latency in escaping onto a hidden platform during 5-day training, suggested that the FA treatment but not vehicle treatment prevented learning deficit in AD mice relative to WT at 7 months old. (**b**) Spatial memory, as indicated by the time spent in the target quadrant (s) during the probe test 24 h after the final training, was impaired in AD mice after the vehicle treatment. This impaired spatial memory was effectively prevented by the FA treatment, to the levels without difference from that in WT mice. (**c**) Mean of the swimming speed during a training trial on the first day was not different among the groups. (**d**) Representative tracking traces during the probe test of the Morris water maze for measuring spatial memory. Data are presented as mean ± SEM. *P < 0.05, **P < 0.01

### FA Prevents Spatial Memory Deficit Completely

Finally, we examined if FA could prevent spatial memory deficit in AD mice from 6 to 7 months old. AD mice with the 30-day FA treatment performed the spatial learning task significantly better than those with the vehicle treatment did, as reflected by a shorter latency in escaping onto a hidden platform during 5-day training (Fig. 6a, WT, *N* = 12; AD or AD-FA, *N* = 13; group, *F*_(2, 35)_ = 4.429, *P* = 0.019; day, *F*_(4, 140)_ = 32.82, *P* < 0.001; group × day, *F*_(8, 140)_ = 1.64, *P* = 0.118; post hoc: WT *vs*. AD, *P = 0.034; WT *vs*. AD-FA: *P* = 0.988; AD *vs*. AD-FA:*P = 0.042. Two-way ANOVA followed by Tukey’s post hoc), suggesting the full restoration of impaired spatial learning by the FA treatment. Probe test 24 h after learning suggested that AD mice with the FA treatment fully restored spatial memory deficit, which was observed in AD mice with the vehicle treatment (Fig. 6b, WT, *N* = 12; AD or AD-FA, *N* = 13; WT *vs*. AD, *P = 0.023; WT *vs*. AD-FA: *P* = 0.972; AD *vs*. AD-FA: *P = 0.011. One-way ANOVA followed by Tukey’s post hoc). These protective effects of FA on impaired spatial learning and memory were not relevant to any changes of swimming speed (Fig. 6c, d, WT, *N* = 12; AD or AD-FA, *N* = 13; WT *vs*. AD, *P* = 0.208; WT *vs*. AD-FA: *P* = 0.298; AD *vs*. AD-FA: *P* = 0.972. One-way ANOVA followed by Tukey’s post hoc).

Taken together, these data strongly suggested that reduced density/diameter of hippocampal capillaries could be also necessary for initiating Aβ plaque deposition and spatial memory deficit in AD mice at the initial stages, as indicated by FA treatment for 30 days prevented reduced density/diameter of hippocampal capillaries and repaired spatial memory deficit completely but Aβ plaque deposition and aggregative microglial cells partially.

## Discussion

**Fig. 7:**
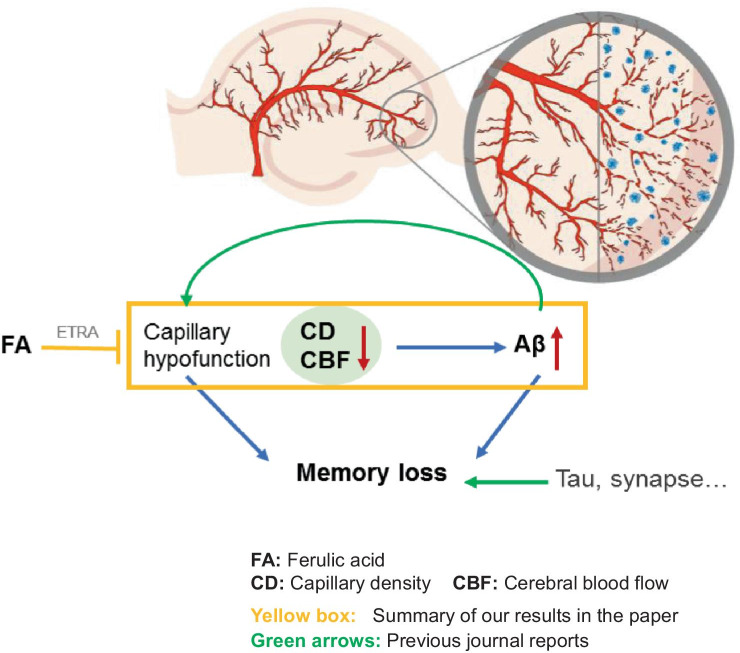
The schematic diagram for a feedforward cycle among capillary, Aβ plaque, and memory loss in AD. Decreased CD/CBF and increased Aβ aggregation could form a feedforward cycle (red and green arrows), leading to memory loss and Aβ plaque deposition that trigger other cascades of the pathophysiology. This feedforward cycle at the initial stages could be antagonized by FA via targeting the ETRA (yellow box)

In the present study, we demonstrate that hypoperfusion of the hippocampus due to reduced density and diameter of capillaries is probably earlier than and crucial for initiating Aβ plaque deposition and spatial memory deficit, providing new evidence supporting a feedforward cycle of the hypoperfusion-Aβ aggregation-more hypoperfusion [10] (Fig. 7) and for understanding why the prevalence of AD is dramatically increased following aging [1].

The earliest sign of memory decline in AD is mostly restricted to the impairment of episodic memories [34], for which the hippocampus plays a crucial role [35]. Studies on the underlying mechanisms should prompt investigation into the therapeutic control of this impairment. Previous studies have suggested that episodic memory deficit in early AD is attributable to ineffective encoding or failed retrieval of memory in AD patients or rodent models [72–75]. Consistent with these findings, we report that spatial learning and memory are both impaired in AD mice at 7 months old, for which reduced density and diameter of hippocampal capillaries are the earliest events. These vascular changes would have caused a tiny hypoperfusion insult, not easily detectable in the hippocampus. We thus mimicked this situation by the injection of ET1 into the hippocampus and found that ET1 induced a small detectable hypoperfusion insult and early Aβ plaque deposition at 5 months old. The cause and the effect may have jointly caused the double-hits [19, 67] on the hippocampal functions of spatial learning and memory. This may have well explained why the Aβ-directed therapies alone have failed in halting or curing memory decline in AD patients [11, 76–78] because hypoperfusion of brain capillaries was not solved. Thus, a combination of the Aβ-directed therapies with those agents targeting brain capillaries could be beneficial for memory decline in AD at the early stages.

An alternative strategy is to prevent hypoperfusion of hippocampal capillaries as early as possible in the aging population. Our data reveal that hypoperfusion of hippocampal capillaries could be also necessary for initiating Aβ plaque deposition and spatial memory deficit because FA treatment for 30 days not only prevents reduced density and diameter of brain capillaries via the ETRA but also repairs spatial memory deficit completely. Consistent with our findings, reduction in the ET-1 levels or RAGE receptor expression has been also reported contributing to the improvement of vascular function. RAGE-dependent BBB transport of circulating Aβ results in the production of ET-1 to cause decreased CBF; infusion of either RAGE-specific IgG, soluble RAGE (as a decoy receptor), or RAGE inhibitor treatment in AD transgenic mice causes an increase in CBF and reduction in brain Aβ levels [79, 80]. The RAGE blocker, FPS-ZM1, has been broadly used in various experimental paradigms in more than 75 follow-up studies. Moreover, small molecule RAGE blockers are currently understudying in the phase 2/3 trial in patients with mild Alzheimer’s and impaired glucose tolerance (ClinicalTrials.gov identifier NCT03980730). Thus, preventing vascular hypofunction is a promising strategy for AD treatment.

Furthermore, a single mechanism of action is hard to produce fully beneficial effects in AD because AD is a multifaceted disease and a combination of several drugs with multiple mechanisms of action is suggested to be essential. Here, we demonstrate that a natural compound FA can produce multiple effects on hippocampal capillaries, spatial memory, Aβ plaque deposition, and aggregative microglial cells. Some earlier preclinical studies also have demonstrated the protective effects of FA or FA based hybrids in AD mouse models. In these studies, FA was found to produce actions such as reducing Aβ deposition or promoting anti-inflammatory or antioxidant effects [37, 69, 71, 81–83]. The present study further strengthened this idea, for which FA not only caused a reduction of Aβ plaques and aggregative microglial cells but also produced protective effects on capillaries possibly via the ETRA, which have not been reported before. It is important to note that reduced density and diameter of hippocampal capillaries could be particularly crucial for AD at the earliest stages. FA intervention later than these stages could be ineffective because Aβ plaque deposition and aggregative microglial cells could have caused other cascades of the pathophysiology of AD [2–10]. Moreover, our RNA sequencing analysis suggested distinct profiles of gene expression for AD mice with the 30-day FA or vehicle treatment relative to WT mice, further supporting multiple effects of FA on brain capillaries and Aβ plaque deposition, and other possible actions (Supplementary Fig. 7). Notably, a recent multicenter, randomized, double-blinded clinical study has investigated the effects of Feru-guard that contained FA for treating mild cognition impairment (MCI) patients aged 65 to 85 years old and the results strongly support the opinion that FA is beneficial for MCI [84], a possible early stage of AD.

It is urgent to find disease-modifying therapy to halt or cure the progressive decline of memory and other cognitive functions in AD. Here, our data provide novel evidence supporting that FA supplement taking as early as possible against the ET1-mediated ETRA activation on hippocampal capillaries is potentially beneficial for memory decline in AD at the earliest stages.

## Supplementary Information

Below is the link to the electronic supplementary material.

Supplementary file1 (PDF 377 KB)

Supplementary file2 (PDF 376 KB)

Supplementary file3 (PDF 380 KB)

Supplementary file4 (PDF 380 KB)

Supplementary file5 (PDF 380 KB)

Supplementary file6 (PDF 380 KB)

Supplementary file7 (PDF 379 KB)

Supplementary file8 (PDF 380 KB)

Supplementary file9 (PDF 295 KB)

Supplementary file10 (PDF 380 KB)

Supplementary file11 (PDF 212 KB)

Supplementary file12 (PDF 213 KB)

Supplementary file13 (PDF 212 KB)

Supplementary file14 (PDF 213 KB)

Supplementary file15 (PDF 212 KB)

Supplementary file16 (PDF 212 KB)

Supplementary file17 (PDF 213 KB)

Supplementary file18 (PDF 463 KB)

Supplementary file19 Figure 1. Ultrastructures associated with hippocampal capillaries. Figure 2. Hippocampal microglia and astrocyte. Figure 3. FA alleviates hypoperfusion insult in the mouse hippocampus. Figure 4. FA targets the ETRA. Figure 5. FA inhibits BACE1 activity. Figure 6. FA reduces aggregative microglial cells. Figure 7. RNA-seq reveals distinct profiles with FA treatment. (DOCX 21937 KB)
