# Supplementary material for: Ferulic Acid Ameliorates Alzheimer’s Disease-like Pathology and Repairs Cognitive Decline by Preventing Capillary Hypofunction in APP/PS1 Mice
Source: Neurotherapeutics. 2021 Mar 30;18(2):1064–80. doi: 10.1007/s13311-021-01024-7 (PMC8423929; doi:10.1007/s13311-021-01024-7)
Supplement: Supplementary file 16 — Supplementary file16 (PDF 212 KB) [file 13311_2021_1024_MOESM16_ESM.pdf]

# ICMJE Form for Disclosure of Potential Conflicts of Interest

## Section 1. Identifying Information

1. Given Name (First Name)
Jinnan

2. Surname (Last Name)
Li

3. Effective Date (07-August-2008)
11-October-2020

4. Are you the corresponding author? ☐ Yes ☑ No

Corresponding Author's Name
Lin Xu

5. Manuscript Title
Ferulic acid against reduced density and diameter of hippocampal capillaries restores Aβ plaque deposition and spatial memory deficit in APP/PS1 mice

6. Manuscript Identifying Number (if you know it)
NERX-D-20-00453

## Section 2. The Work Under Consideration for Publication

Did you or your institution at any time receive payment or services from a third party for any aspect of the submitted work (including but not limited to grants, data monitoring board, study design, manuscript preparation, statistical analysis, etc…)?

Complete each row by checking "No" or providing the requested information. If you have more than one relationship click the "Add" button to add a row. Excess rows can be removed by clicking the "X" button.

**The Work Under Consideration for Publication**

| Type | No | Money Paid to You | Money to Your Institution* | Name of Entity | Comments** | |
|---|---|---|---|---|---|---|

* This means money that your institution received for your efforts on this study.
** Use this section to provide any needed explanation.

## Section 3. Relevant financial activities outside the submitted work.

Place a check in the appropriate boxes in the table to indicate whether you have financial relationships (regardless of amount of compensation) with entities as described in the instructions. Use one line for each entity; add as many lines as you need by clicking the "Add +" box. You should report relationships that were present during the 36 months prior to submission.

Complete each row by checking "No" or providing the requested information. If you have more than one relationship click the "Add" button to add a row. Excess rows can be removed by clicking the "X" button.

**Relevant financial activities outside the submitted work**

ICMJE INTERNATIONAL COMMITTEE of MEDICAL JOURNAL EDITORS

# ICMJE Form for Disclosure of Potential Conflicts of Interest

**Relevant financial activities outside the submitted work**

| Type of Relationship (in alphabetical order) | No | Money Paid to You | Money to Your Institution* | Entity | Comments | |
|---|---|---|---|---|---|---|
| Type of Relationship (in alphabetical order) | No | Money Paid to You | Money to Your Institution* | Entity | Comments | |

* This means money that your institution received for your efforts.
** For example, if you report a consultancy above there is no need to report travel related to that consultancy on this line.

## Section 4. Other relationships

Are there other relationships or activities that readers could perceive to have influenced, or that give the appearance of potentially influencing, what you wrote in the submitted work?

☑ No other relationships/conditions/circumstances that present a potential conflict of interest

☐ Yes, the following relationships/conditions/circumstances are present (explain below):

At the time of manuscript acceptance, journals will ask authors to confirm and, if necessary, update their disclosure statements. On occasion, journals may ask authors to disclose further information about reported relationships.

Show All Table Rows    SAVE

## Evaluation and Feedback

Please visit http://www.icmje.org/cgi-bin/feedback to provide feedback on your experience with completing this form.
