# Supplementary material for: Ferulic Acid Ameliorates Alzheimer’s Disease-like Pathology and Repairs Cognitive Decline by Preventing Capillary Hypofunction in APP/PS1 Mice
Source: Neurotherapeutics. 2021 Mar 30;18(2):1064–80. doi: 10.1007/s13311-021-01024-7 (PMC8423929; doi:10.1007/s13311-021-01024-7)
Supplement: Supplementary file 19 — Supplementary file19 Figure 1. Ultrastructures associated with hippocampal capillaries. Figure 2. Hippocampal microglia and astrocyte. Figure 3. FA alleviates hypoperfusion insult in the mouse hippocampus. Figure 4. FA targets the ETRA. Figure 5. FA inhibits BACE1 activity. Figure 6. FA reduces aggregative microglial cells. Figure 7. RNA-seq reveals distinct profiles with FA treatment. (DOCX 21937 KB) [file 13311_2021_1024_MOESM19_ESM.docx]

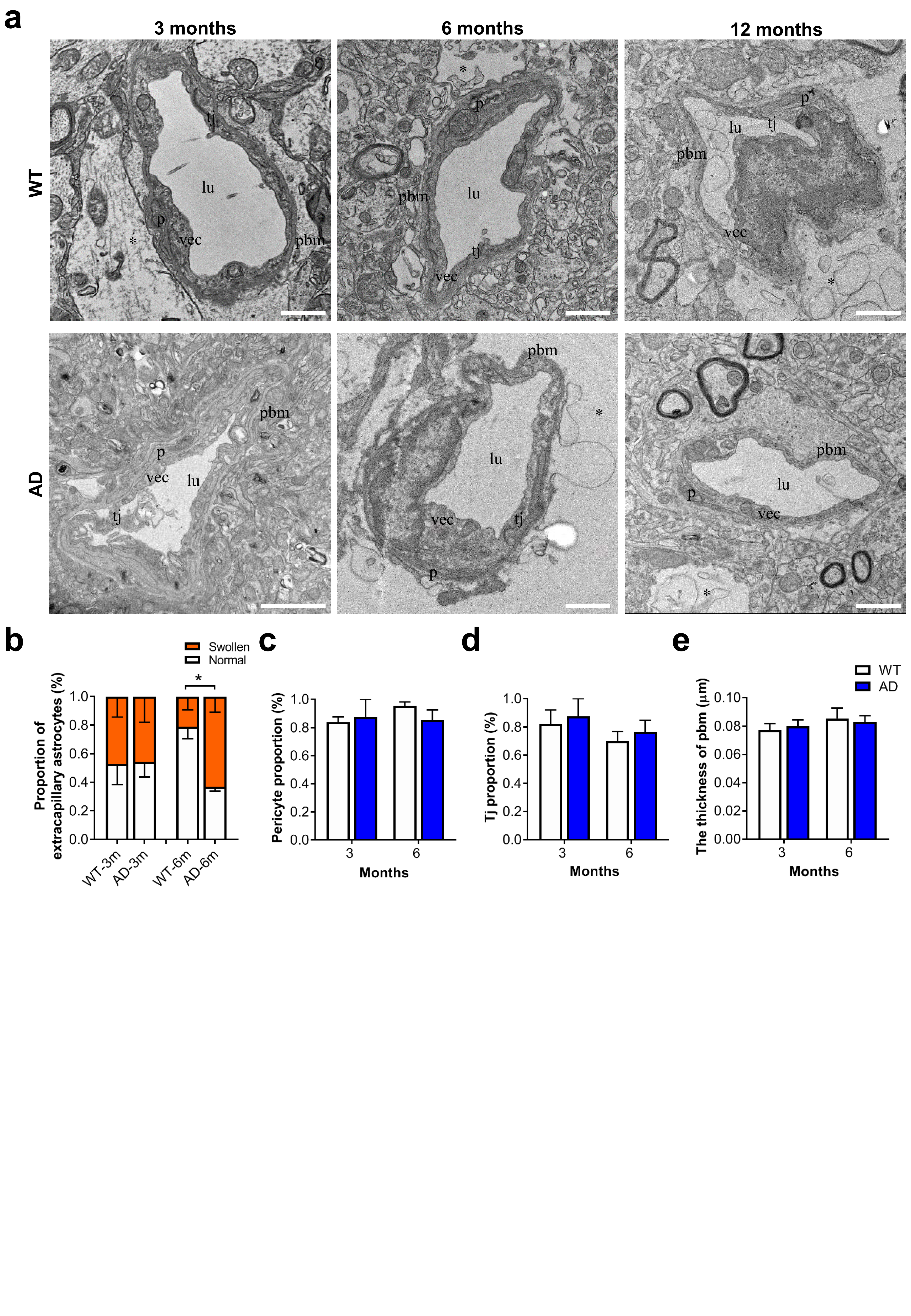


**Figure 1.** Ultrastructures associated with hippocampal capillaries. **a,** Representative images for hippocampal capillary using transmission electron microscopy. **b**, The proportion of the extra-capillary swollen astrocytes was higher in AD mice than in WT at 6-month-old (3-month-old: WT, N = 3; AD, N = 4; *t* = 0.056, *df* = 5, *P* = 0.957. 6-month-old: N = 4/group；*t* = 2.921, *df* = 6, **P* = 0.026; student’s *t*-test). **c**, The pericyte (p) proportion was not different between AD and WT mice at 3 or 6-month-old (3-month-old: WT, N = 3; AD, N = 4; *P* = 0.765. 6-month-old: n = 4/group; *P* = 0.625). **d**, The tight junction (Tj) proportion was not different between AD and WT mice at 3 or 6-month-old (3-month-old: WT, N = 3; AD, N = 4; *P* = 0.859. 6-month-old: N = 4/group; *P* = 0.859). **e**, The thickness of the perivascular basement membrane (pbm) was not different between AD and WT mice at 3 or 6-month-old (3-month-old: WT, N = 3; AD, N = 4; *P* = 0.929. 6-month-old: N = 4/group; *P* = 0.929). Two-way ANOVA followed by Holm-Sidak's multiple comparison test in **c-e**. Data are presented as mean ± s.e.m. **P* < 0.05. lu = lumen; vec = vascular endothelial cell.

**Fig****ure 2.** Hippocampal microglia and astrocyte. **a**, Immunostaining of hippocampal microglia using the antibody *Iba1*. **b**, The total number of microglial cells was not different at 4 month-old but increased at 7 or 9 month-old in AD mice relative to WT mice (4-month-old: WT *vs*. AD, *P* = 0.999; 7-month-old: WT *vs*. AD, ***P* = 0.001; 9-month-old: WT *vs*. AD, ****P* < 0.001) **c,** The aggregative microglia area (%) was significant larger in AD mice than in WT mice since 7 or 9-month-old (4-month-old: WT *vs.* AD, *P* = 0.946; 7-month-old: WT *vs*. AD, ****P* < 0.001; 9-month-old: WT vs. AD, ****P* < 0.001). **d**, Immunostaining of hippocampal astrocyte using the antibody *GFAP*. **e**, GFAP^+^ area (%) suggested no difference between AD and WT mice at 4 or 7-month-old but was larger in AD than in WT mice at 9-month-old (4-month-old: WT *vs.* AD, *P* = 0.774; 7-month-old: WT *vs*. AD, *P* = 0.774; 9-month-old: WT *vs*. AD, ****P* < 0.001). For **b**, **c**, and **e**, 4 or 7-month-old: WT or AD, n = 17-18 (N = 3); 9-month-old: WT, n = 17-18 (N = 3); AD, n = 24 (N = 4); two-way ANOVA followed by Holm-Sidak’s analysis. **f-g**, DAB immunolabeling of hippocampal microglia also suggested that the total number of microglial cells was increased at 7-month-old in AD mice relative to WT mice (WT, n = 18 (N = 3); AD, n =18 (N = 3); *t* = 3.591, *df* = 34, ***P* = 0.001). Data are presented as mean ± s.e.m. ***P* < 0.01, ****P* < 0.001.


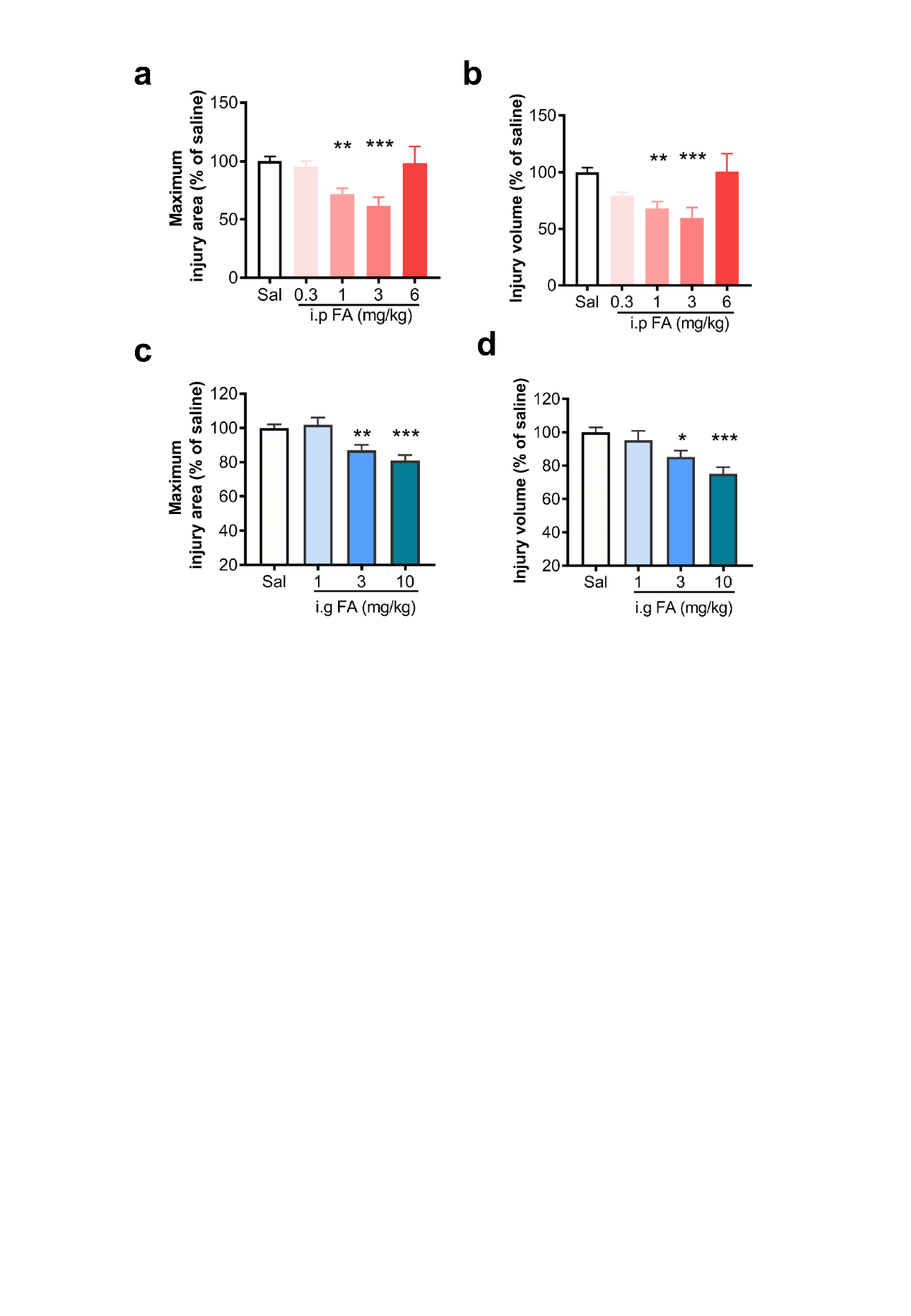
**Figure 3.** FA alleviates hypoperfusion insult in the mouse hippocampus. **a**, **b**, In a photo-thrombosis mouse model of acute ischemic stroke (AIS) in the hippocampal CA1, mice received FA (i.p.) and the infarction was measured 24 h after AIS induction. FA alleviated the maximum injury area (**a**) or the total injury volume (**b**) relative to the Sal control (Sal, N =18; 0.3 mg/kg, N = 4; 1 mg/kg, N = 12; 3 mg/kg, N = 8; 6 mg/kg, N = 5; *Maximum injury area*: Sal *vs.* 0.3 mg/kg, *P* = 0.977; Sal *vs*. 1 mg/kg, ***P* = 0.001; Sal *vs*. 3 mg/kg, ****P* < 0.001; Sal *vs*. 6 mg/kg, *P* = 0.999. *Injury volume*: Sal vs. 0.3 mg/kg, *P* = 0.257; Sal *vs*.1 mg/kg, ***P* = 0.001; Sal *vs*. 3 mg/kg, ****P* < 0.001; Sal *vs*. 6 mg/kg, *P* = 0.999). **c**, **d**, Intragastric administration (i.g.) of FA similarly alleviated the maximum injury area (**c**) or the total injury volume (**d)** relative to the Sal control (Sal, N = 86; 1 mg/kg, N = 27; 3 mg/kg, N = 42; 10 mg/kg, N = 46; *Maximum injury area*: Sal *vs*. 1 mg/kg, *P* = 0.951; Sal *vs*. 3 mg/kg, ***P* = 0.003; Sal vs. 10 mg/kg, ****P <* 0.001. *Injury volume*: Sal vs. 1 mg/kg, *P* = 0.783; Sal *vs*. 3 mg/kg, **P* = 0.012; Sal *vs*. 10 mg/kg, ****P* < 0.001). One-way ANOVA was used for statistical analysis. Data are presented as mean ± s.e.m. **P* < 0.05, ***P* < 0.01, ****P* < 0.001.


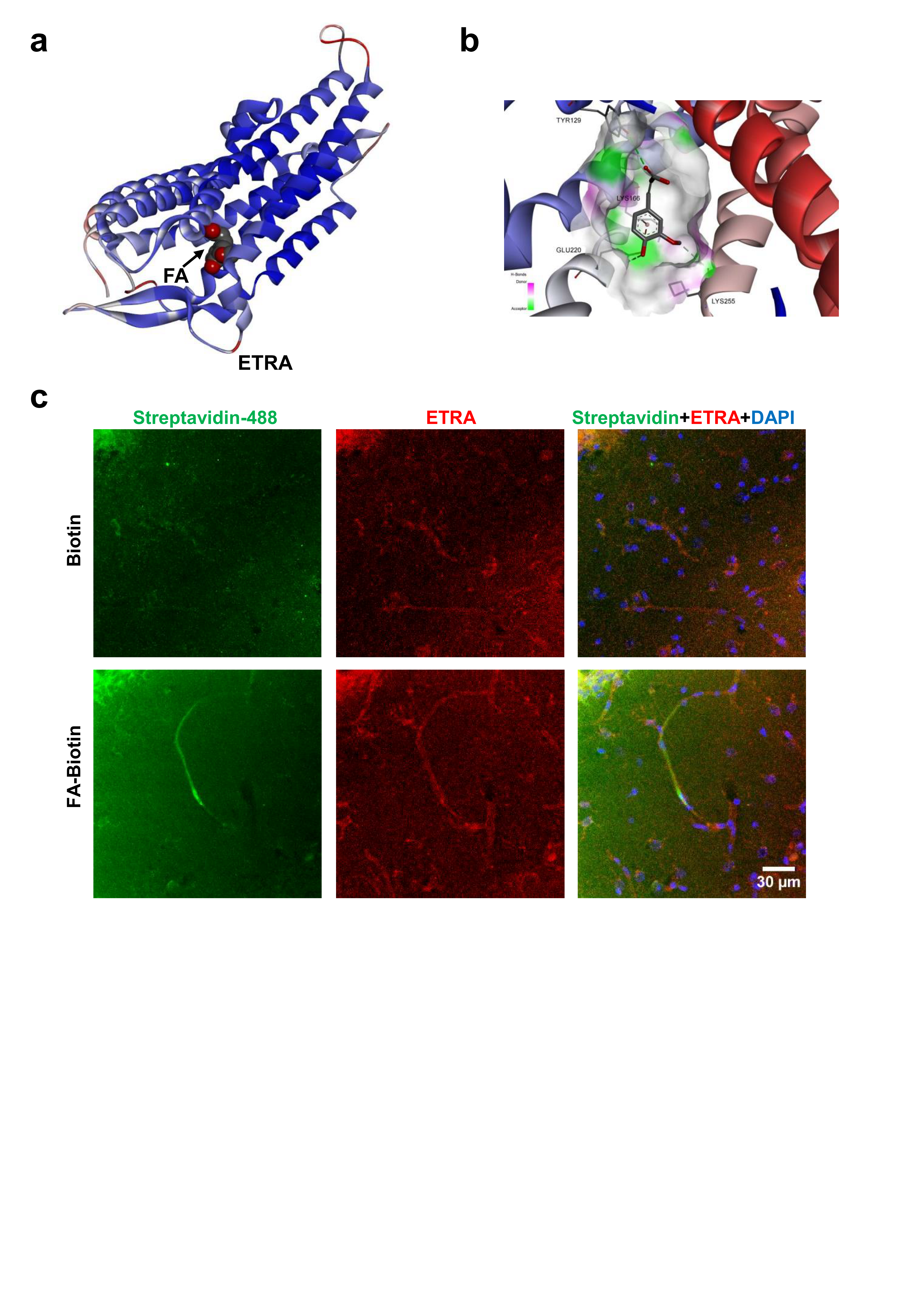


**Figure 4.** FA targets the ETRA. **a**, Molecular docking experiment suggested that FA had a high-affinity binding to the ETRA (-5.3 Kcal/mol). **b**, Four amino acid residues of the ETRA bound to FA *via* hydrogen-bond interaction. **c**, Mice recived FA-biotin, and immunostaining of FA-biotin (streptavidin-488, *green*) and ETRA (*red*) suggested co-localization of FA and ETRA mainly on the blood vessels of the hippocampus. ETRA = the endothelin receptor A.


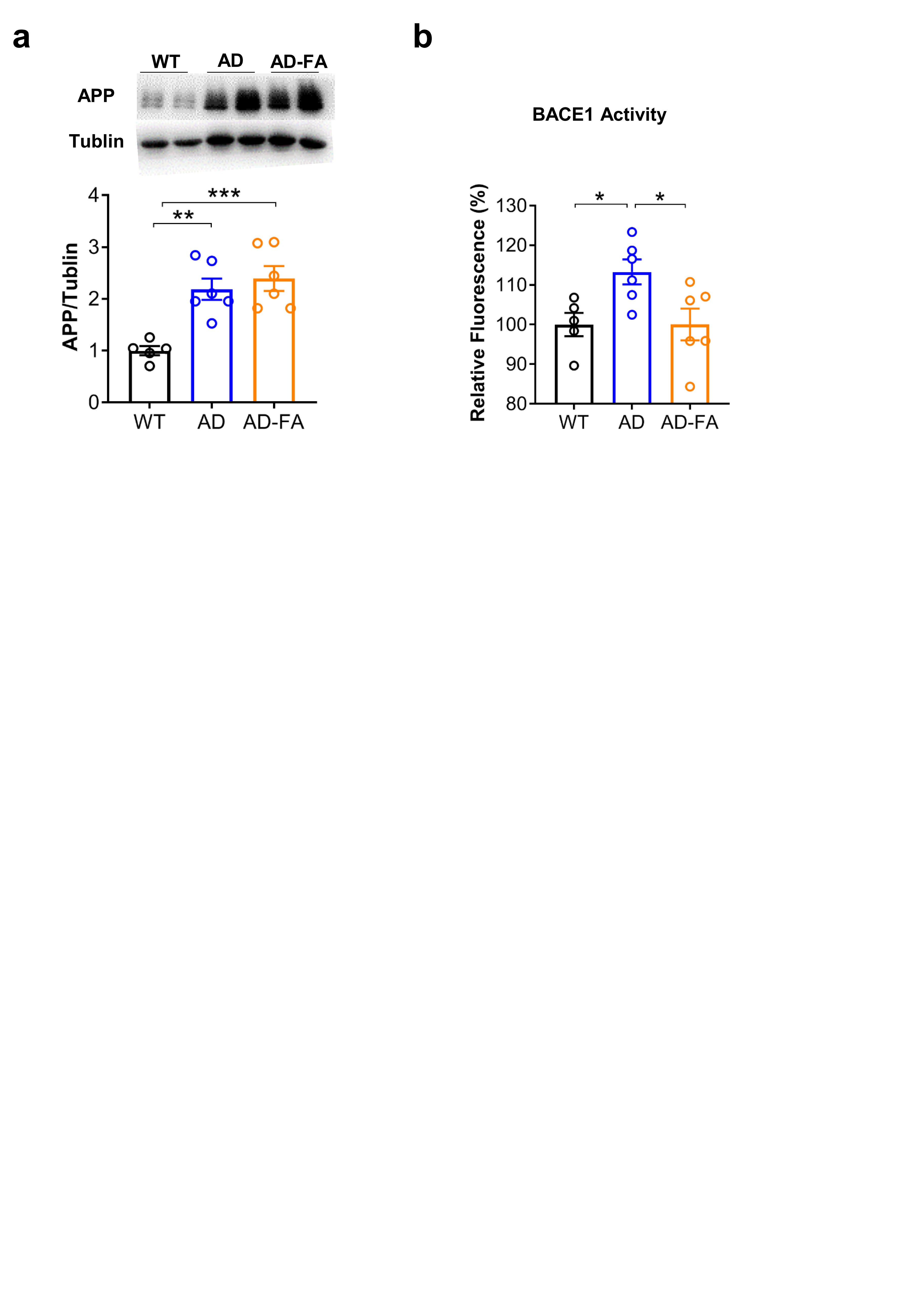


**Figure 5.** FA inhibits BACE1 activity. **a**, Expression of the APP was higher in AD mice (AD or AD-FA) than in WT mice, while FA treatment (AD-FA) had no effect on APP expression as compared with the vehicle control (AD) (WT, N = 5; AD or AD-FA, N = 6; *F* _(2, 14)_ = 13.35, *P* < 0.001; WT *vs*. AD: ***P* = 0.002; WT *vs*. AD-FA: ****P* < 0.001; AD *vs*. AD-FA: *P* = 0.736). **b**, Relative fluorescence indicated that BACE1 activity was increased with the vehicle treatment, but that was restored to the WT level by the FA treatment (WT, N = 5; AD or AD-FA, N = 6; *F* _(2, 14)_ = 5.028, *P* = 0.022; WT *vs*. AD: **P* = 0.046; WT *vs*. AD-FA: *P* = 0.999; AD *vs*. AD-FA: **P* = 0.036). One-way ANOVA followed by Tukey's Post hoc. Data are presented as mean ± s.e.m. **P* < 0.05, ***P* < 0.01, ****P* < 0.001.

**Figure 6.** FA reduces the area of aggregative microglial cells. **a**, Co-immunostaining of microglia (*Iba1*) and Aβ plaque (*D54D2*) in the hippocampus from AD or WT mice with the FA (AD-FA) or vehicle (AD) treatment for 30 days, at 7-month-old. **b**, The total number of the microglial cells shown as the Iba1^+^ suggested no differences between the AD and AD-FA groups (AD, n = 25 (N = 3); AD-FA, n =28 (N = 4); *t* = 1.14, *df* = 51, *P* = 0.259). **c**, The area of aggregative microglial cells (%) was decreased after the FA treatment relative to the vehicle control (AD, n = 25 (N = 3); AD-FA, n =28 (N = 4); *t* = 3.167, *df* = 51, ***P* = 0.002). **d,** Immunostaining of astrocyte (*GFAP*) in the hippocampus and the cortex from AD or WT mice after the FA or vehicle treatment for 30 days, at 7-month-old. **e**, **f**, GFAP^+^ area (%) in the hippocampus (**e**) or the cortex (**f**) was not different after the FA treatment relative to the vehicle control (*Hippocampus*: AD, n = 18 (N = 3); AD-FA, n =24 (N = 4); *t* = 0.596, *df* = 40, *P* = 0.554; *Cortex*: AD, n = 18 (N = 3); AD-FA, n =22 (N = 4); *t* = 1.596, *df* = 38, *P* = 0.118). Student’s *t*-test. Data are presented as mean ± s.e.m. ***P* < 0.01.


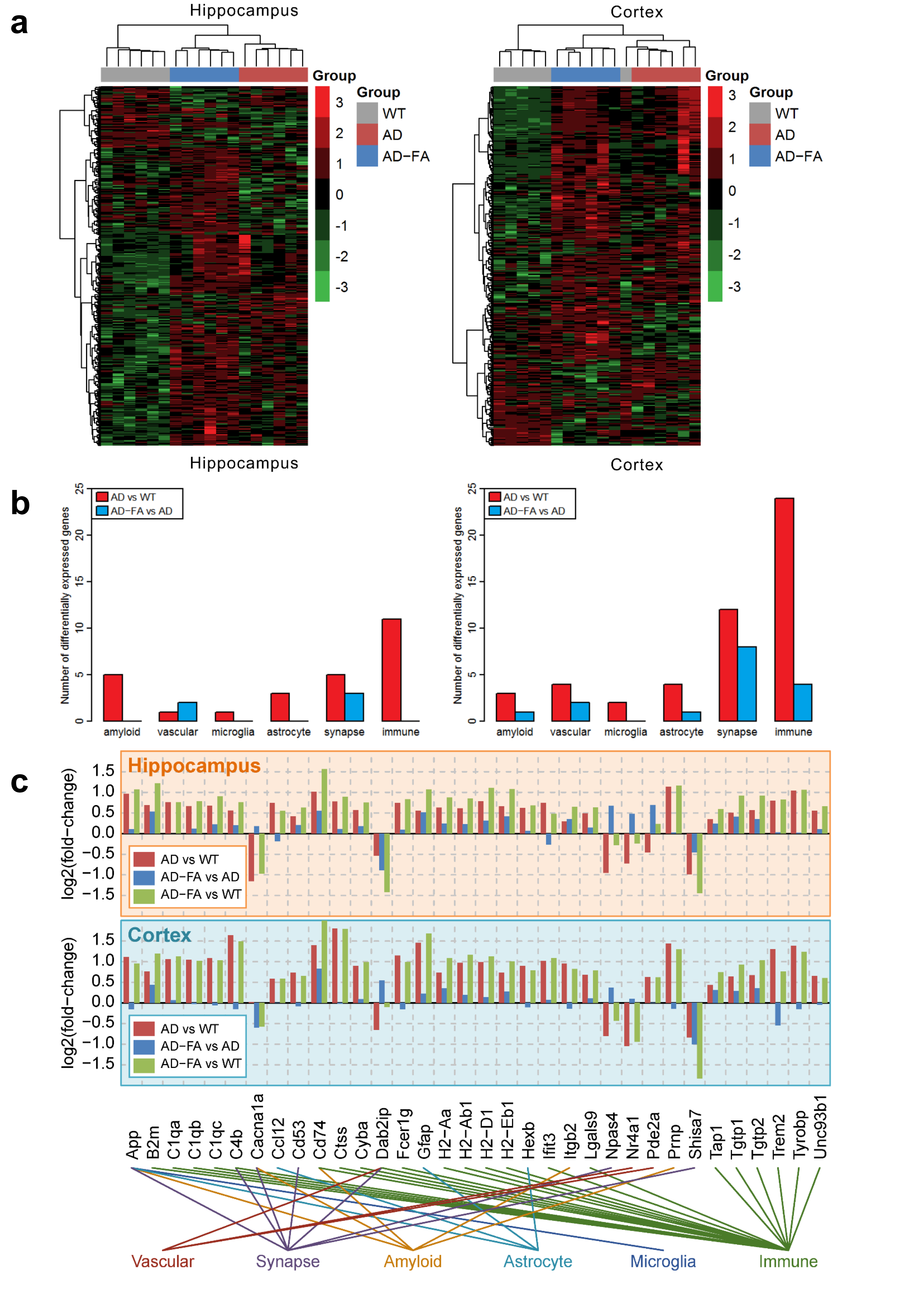


**Figure 7.** RNA-Seq reveals distinct profiles with the FA treatment. **a**, Heatmap and hierarchical clustering of differentially expressed genes in the hippocampus or the cortex from AD mice with vehicle (AD or WT) or FA treatment (AD-FA) for 30 days. All gene expression values were z-score converted. **b**, The number of differentially expressed genes in specified biological functions. **c**, The fold-change of differentially expressed genes in specified biological functions shared by the hippocampus and the cortex.
